# Evidence base and practice variation in acute care processes for knee and hip arthroplasty surgeries

**DOI:** 10.1371/journal.pone.0180090

**Published:** 2017-07-19

**Authors:** Marcel Mayer, Justine Naylor, Ian Harris, Helen Badge, Sam Adie, Kathryn Mills, Joseph Descallar

**Affiliations:** 1 Whitlam Orthopaedic Research Centre, Liverpool Hospital, Sydney, New South Wales, Australia; 2 South Western Sydney Clinical School, UNSW, Randwick, Australia; 3 Technical University Munich, Munich, Bavaria, Germany; 4 Ingham Institute for Applied Medical Research, Liverpool, Australia; 5 Macquarie University, North Ryde, Australia; Universita degli Studi di Firenze, ITALY

## Abstract

**Background:**

Lack of evidence contributes to unnecessary variation in treatment costs and outcomes. This study aimed to identify from interventions historically used for total knee or hip arthroplasty (TKA, THA): i) if routine use is supported by high-level evidence; ii) whether surgeon use aligns with the evidence.

**Methods:**

Part 1: Systematic search of electronic library databases for systematic reviews and practice guidelines concerning seven acute-care interventions. Intervention-specific recommendations concerning routine use were extracted by assessors. Part 2: Prospective medical record audit of the acute-care received by 1900 patients involving 120 orthopaedic surgeons. Surgeon use per intervention was summarized using caterpillar plots. Surgeon-specific routine and non-routine use was defined as use in ≥ 90% and ≤ 10% of patients, respectively. Primary analysis included only surgeons contributing ≥ 10 patients.

**Results:**

*Continuous passive motion (TKA)*: Routine use not recommended; 85.7% of surgeons did not use it routinely. *Tranexamic Acid*: Routine use recommended; 26.9% of surgeons used it routinely. *Cryotherapy*: Routine use not recommended; 45.7% of surgeons used it routinely for TKA; 31.8% used it routinely for THA. *Intra-articular drainage*: Routine use not recommended for TKA, but possible benefits for THA; 5.7% of surgeons used it routinely for TKA, 0.0% used it routinely for THA. *Antibiotic loaded bone cement*: Routine use for TKA not supported, recommendations for use for THA are inconsistent; 90.0% of surgeons used it routinely for TKA, 100.0% used it routinely for THA. *Patella resurfacing (TKA)*: No recommendation could be made; 57.1% of surgeons routinely resurfaced the patella. *Indwelling urinary catheterisation*: Routine use recommended; 59.6% of surgeons used it routinely.

**Conclusion:**

Recommendations for routine use or not exist for some of the acute-care interventions examined. Surgeon practices vary widely even in the presence of high-level recommendations. It is unclear whether further evidence alone would lessen unwarranted practice variation.

## Introduction

Total knee arthroplasty (TKA) and total hip arthroplasty (THA) surgeries are considered to be highly cost-effective treatments for end-stage osteoarthritis [1]. The surgeries significantly improve pain, mobility, function, and quality of life for most people who undergo them in both the short- and long-term [2–4]. Given their success and the aging population, the volume of these surgeries is increasing globally [5–9].

TKA and THA surgeries lend themselves to standardized care pathways during the acute-care period [10], as there are particular interventions which need to be routinely considered. These interventions include the use of intravenous antibiotics peri-operatively for the prevention of joint infection and the use of chemoprophylaxis for the prevention of venous thromboembolism (VTE). Infection and VTE prophylaxes are considered mandatory to the arthroplasty pathway given the significant consequences associated with such outcomes. As such, guidelines exist to inform practice [11, 12]. However, there are other care processes in the pathway that address other aspects of recovery which are arguably not as critical. These typically have not been addressed by guidelines despite the existence of high-level evidence regarding their use. These include, but are not limited to, the use of cryotherapy or intra-articular drainage for the attenuation of swelling and pain, and the use of antifibrinolytic agents to reduce local bleeding [13]. A lack of guidelines to inform the appropriate use of various interventions may contribute to inconsistencies in care delivery amongst surgeons. These, in turn, can lead to unnecessary variation in costs associated with care and may contribute to unnecessary differences in outcomes between patients. For these reasons, it is important to identify which interventions should or should not be routinely provided based on the best available evidence, and whether surgeon practices align with the evidence.

This study aimed to identify from seven interventions historically used for TKA or THA: i) those for which routine use is supported or not supported by high-level evidence, and; ii) whether surgeon use aligns with the high-level evidence.

## Methods

The study was conducted in two parts.

### Part 1 –Systematic literature search

Part 1 summarized the available, high-level evidence for seven interventions. The processes selected were based on their relevance to arthroplasty care pathways [10, 13], our own clinical and research expertise in the area, and what data were feasible for hospitals to provide in the prospective audit of patient-level data (see Part 2). Regarding the latter, interventions that required more than a ‘yes’ or ‘no’ answer (for example, specific information related to multimodal analgesic approaches or multi-disciplinary pre-admission processes) were not included. Extensive information about VTE and infection prophylaxes was collected, but is not included here as it forms the basis of a separate study. High-level evidence included systematic reviews and practice guidelines.

Systematic searches were conducted in April 2016 using PubMed/Medline and EMBASE electronic library databases. The interventions included continuous passive motion (CPM) therapy, tranexamic acid (TXA), cryotherapy, intra-articular drainage, antibiotic impregnated cement, patellar resurfacing, and indwelling urinary catheterisation (IDC). Detailed rationale for each intervention are provided in S1 Appendix.

The Medical Subject Headings *Arthroplasty*, *knee* and *Arthroplasty*, *hip* were combined with *meta-analysis*, *review* or *guideline* and a specific key term for each process. The specific terms included *continuous passive motion*, *tranexamic acid*, *cryotherapy*, *drainage*, *antibiotic cement*, *patellar resurfacing*, *indwelling catheter*, *catheterization*, *urinary catheter*, *and bladder catheter*. All the terms for the interventions were exploded when used in Medline and EMBASE [refer to the Fig A in S1 Appendix for an exemplary search strategy for “cryotherapy”]. Whilst no date restriction was applied to the search strategy, to ensure the recommendations found were contemporaneous, only reviews or guidelines published between January 2010 and April 2016 were retrieved. If no recent review could be found, the latest available source of high-level evidence prior to 2010 was used. Only reviews of randomised trials were eligible; narrative reviews, i.e. reviews without a systematic literature search, were excluded. No attempt was made to include or exclude the source based on the quality of the review or guideline, however, an assessment of the methodological quality of the systematic reviews was undertaken using AMSTAR (assessment of multiple systematic reviews) [14]. For each intervention, two authors assessed each included SR; any discrepancies between the scores were resolved through discussion. No restrictions were placed on the language or origin of the included sources.

Three assessors independently summarised the literature for each intervention and extracted the main conclusions from the articles. Each assessor determined whether the intervention was routinely or not routinely supported by the source or whether no conclusion about routine use was or could be made. Once all recommendations for use for each intervention were assembled, group consensus determined the final recommendations, informed by the summaries provided by each assessor. The summaries for each intervention were synthesised and are provided in S1 Appendix.

### Part 2 –Prospective audit

Part 2 involved a prospective audit of acute-care practices of 120 surgeons from 19 arthroplasty hospitals (10 public, nine private) across Australia.

This study was nested within a larger, observational study investigating the relationships between the acute care received and outcomes after primary TKA or THA in people with osteoarthritis. A part-random, part-convenience sample of high-volume arthroplasty hospitals (performing > 275 arthroplasties per year in 2012) listed in the National Joint Replacement Registry participated in the study. Ethical approval was provided by several Human Research Ethics Committees—Hunter New England HREC (NSW); St Vincent's Health and Aged Care HREC (Queensland); Austin Health HREC (Victoria); Barwon Health HREC (Victoria); Epworth HREC (Victoria); Calvary Health Care Clinical and Research Ethics Committee (Tasmania and Riverina) and; Calvary Healthcare Adelaide HREC (South Australia). All patient participants provided informed, written consent pre-operatively. Over a 16-month period (August 2013 to December 2014), 3285 people were screened; 2529 met the inclusion criteria of having a primary diagnosis of osteoarthritis, not planning another arthroplasty procedure within three months of the first, able to be followed-up by telephone, and ability to comprehend the study. Consent was obtained from 1900 people– 1072 undergoing TKA and 828 undergoing THA. Baseline participant information and acute-care length of stay were also collected.

Site coordinators from each hospital completed a study pro forma. The pro forma required information to be extracted from the medical record concerning the surgery, who the surgeon was, and the use or not of the interventions under review. In addition, for two care processes (CPM and cryotherapy), sites were asked to specify when a surgeon used it for a specific indication in an individual patient.

For quality control purposes, all data from medical records were re-abstracted by research staff and the accuracy of data entry was checked in duplicate.

#### Data-analysis

Surgeon-level variation for each process was described using frequency of use (proportion of cases) and the associated 95% confidence interval [15], (Clopper-Pearson (exact) CI) using RStudio V1.0.136. Caterpillar plots were subsequently derived to illustrate the level of consistency or variation between surgeons for each intervention. Surgeons who only contributed one patient to the dataset of interest were excluded from the surgeon-specific analysis. This was because their inclusion would inflate practice uniformity given no variation is possible with a sample of one. As each surgeon contributed a varying number of patients to the dataset, we report the results inclusive of surgeons providing at least 10 patients per analysis as well as reporting the results inclusive of all surgeons providing at least two. We did this because surgeons contributing small numbers will demonstrate wide confidence intervals simply because of their small sample. In addition, we also report the proportion of patients who received each intervention. Depending on the process, the cohort was either analysed as one sample or by joint. Due to incomplete record keeping at hospital level, not all patients had data available for all processes.

To determine whether between-surgeon and between hospital variations were significant, intraclass correlations (ICC) were estimated using 3-level multilevel models with random intercepts, using patient as level 1, surgeon as level 2 and hospital as level 3. In effect the ICCs would reflect what proportion of variation in interventions was attributable to between surgeon differences, and what proportion of the surgeon variation was attributable to between hospital differences. ICCs range from 0 to 1 with higher ICC indicating relatively higher variation between surgeons and between hospitals.

## Results

**Fig 1:**
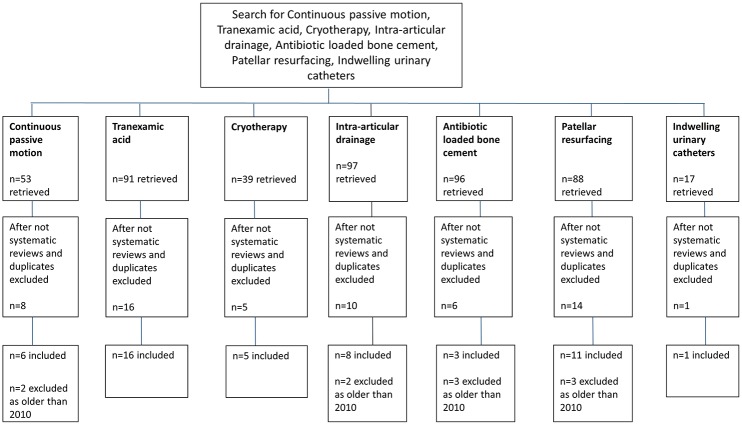
Search strategy for systematic reviews. Each column shows the numbers of systematic reviews included and excluded per step of literature search.

Fig 1 summarizes the number of included and excluded studies per care process found through the systematic search of the literature. Forty-eight SRs and one practice guideline were included.

**Fig 2:**
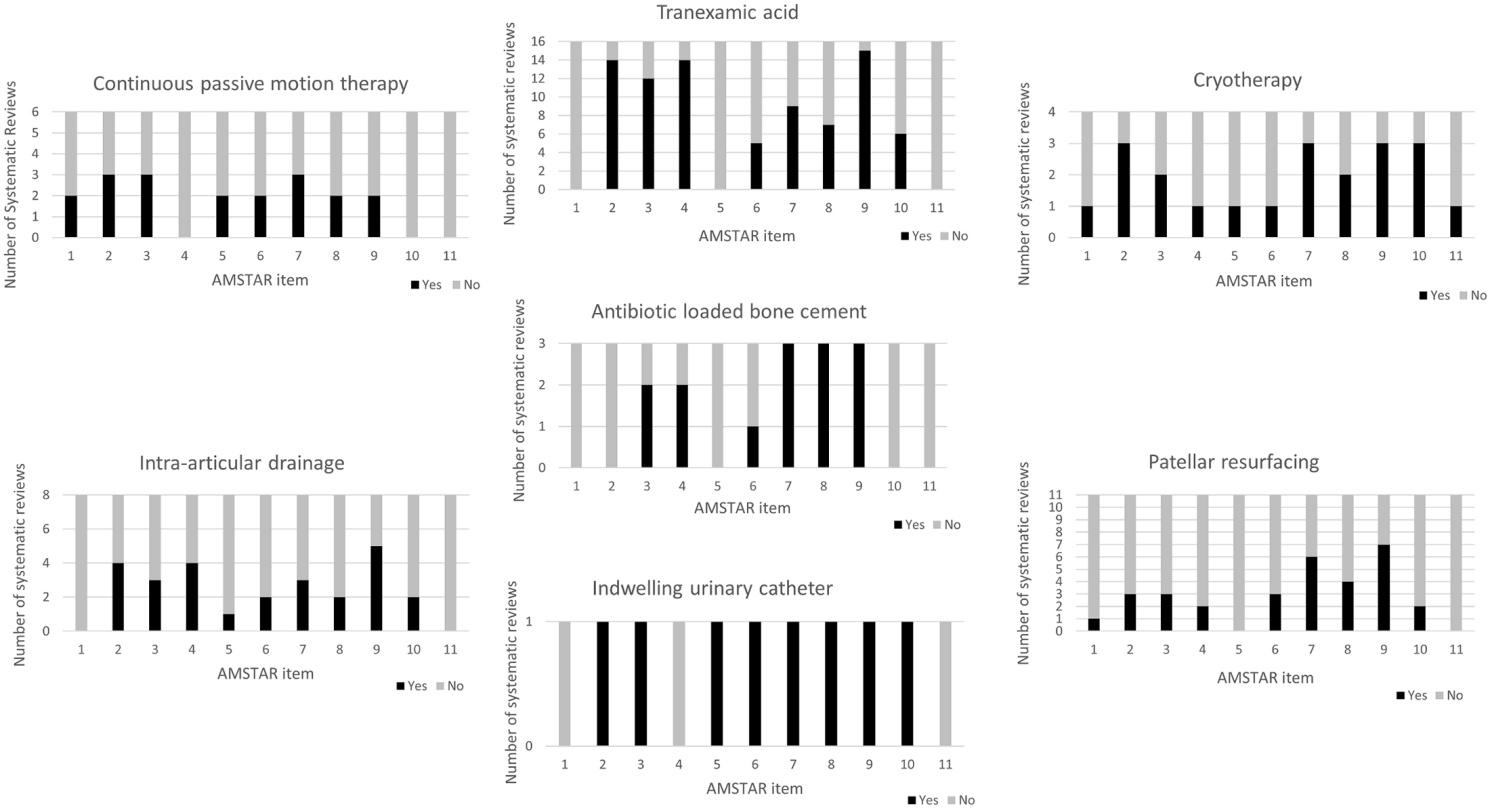
AMSTAR ratings for treatments reviewed. For each process one graph in the multi-panel figure was created. The X-axis of the included figures shows AMSTAR items one to eleven. The Y-axis shows the number of included systematic reviews per process. The black bar indicates how many systematic reviews scored a “YES” per item while the grey bar indicated the number of “NOs”.

A summary of the AMSTAR item scores per care process is provided in Fig 2. Five SRs were not available in English, thus no AMSTAR rating was applied for these though the recommendations provided in the Abstracts (written in English) were included in the main results. Items 3 (questioning if a comprehensive literature search was performed), 7 (questioning if the scientific quality of included reviews was documented) and 9 (questioning if appropriate methods were used to combine results of the studies) were the best performing items whilst items 1 (questioning if the research question was stated before conduct of the review), 5 (questioning if a complete list of included and excluded studies was provided) and 11 (questioning if a potential “conflict of interest” was stated for each study) were the worst performing. Individual AMSTAR total scores per review ranged from 0 to 11. With one exception (the SR for cryotherapy [16]), no other SR scored the maximum (11) points.

For Part 2, the characteristics of the patient sample included in the prospective audit are summarised in Table 1.

**Table 1:** Patient cohort characteristics.

Characteristic	TKA, n = 1072	THA, n = 828
Age, yr	68.4 (8.8)	65.6 (10.9)
Male gender	44%	47%
Body mass index	32.2 (6.7)	29.2 (5.8)
Public insurance status (Medicare)	50%	36%
Comorbidity, 1 or more	93%	86%
American Society of Anesthesiology Score		
1	8%	15%
2	53%	59%
3	34%	25%
4	2%	1%
unknown	3%	
General anaesthetic only	13%	31%
Spinal +/- other	47%	44%
Oxford Knee or Hip Score (maximum/best score = 48)	21.6 (8.2)	20.9 (9.1)
Length of stay, acute ward	6.1 (2.6)	5.1 (2.4)

values are mean (sd) or percentages

**Fig 3:**
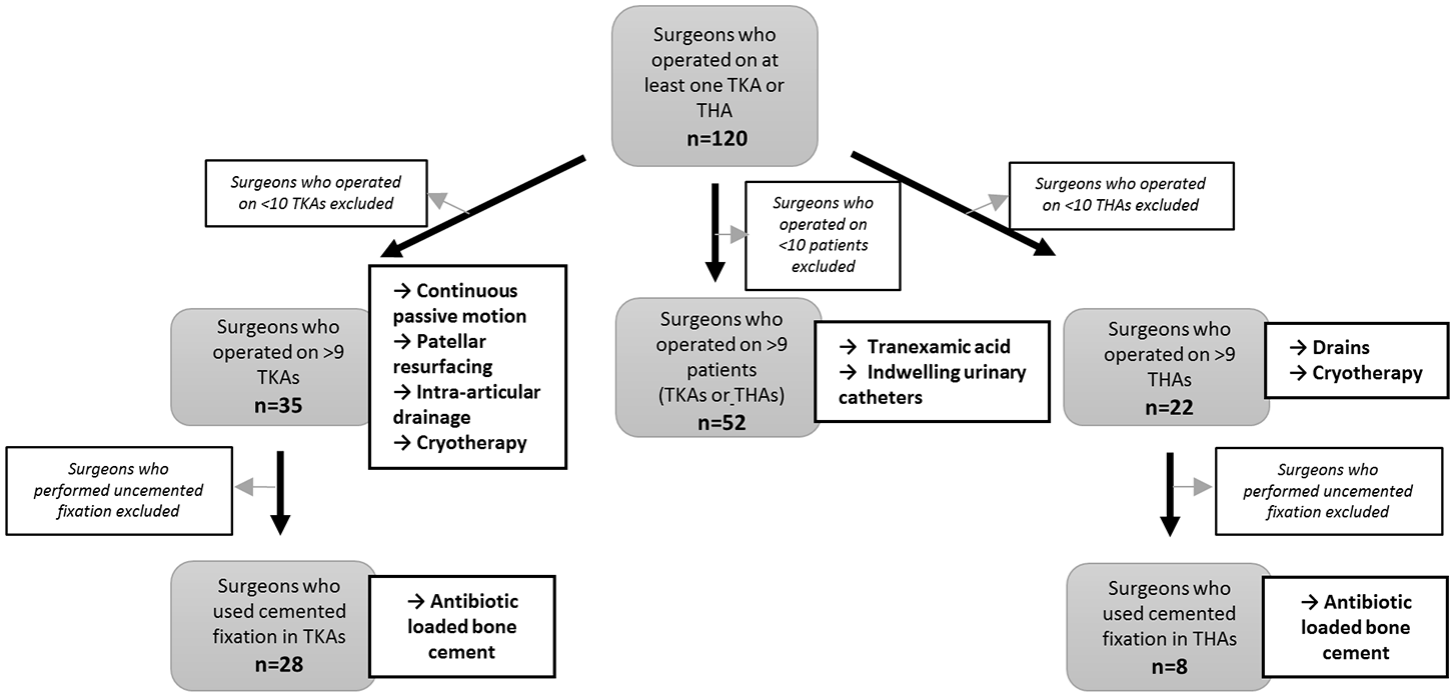
Flow diagram of surgeons. Diagram shows flow of smaller surgeon sample through the study. The smaller sample is object of Results and Discussion.

There were 109 surgeons who performed TKA and 96 surgeons who performed THA. Most (70.1%) performed both surgery types. Removing those who only contributed one patient to the dataset left 86 surgeons for inclusion in the surgeon-specific analysis for TKA and 76 for the THA analysis. The plots including the larger sample of surgeons are provided in the S1 Appendix. Removing those who contributed less than ten patients to the dataset left 8 to 52 surgeons in the sample, depending on the intervention (Fig 3).

### Continuous passive motion

Continuous passive motion (CPM) therapy, applied by a motorized device, passively moves the limb over a predefined range of motion [17]. It potentially provides a positive effect on rehabilitation [18, 19], but has been questioned due to the cost and the requirement for the patient to be in bed.

#### Recommendations from the literature

Two systematic reviews, three meta-analyses and one guideline were found [20–25] (Table A in S1 Appendix)). All six reviews concluded that there is no justification for the routine use of CPM following TKA for optimization of knee flexion or extension, quality of life or the reduction of length of stay (LOS) during the acute and sub-acute period. Mean AMSTAR score for the included reviews was 43% (range 0–73%).

#### Surgeon practice—High volume cohort

**Fig 4:**
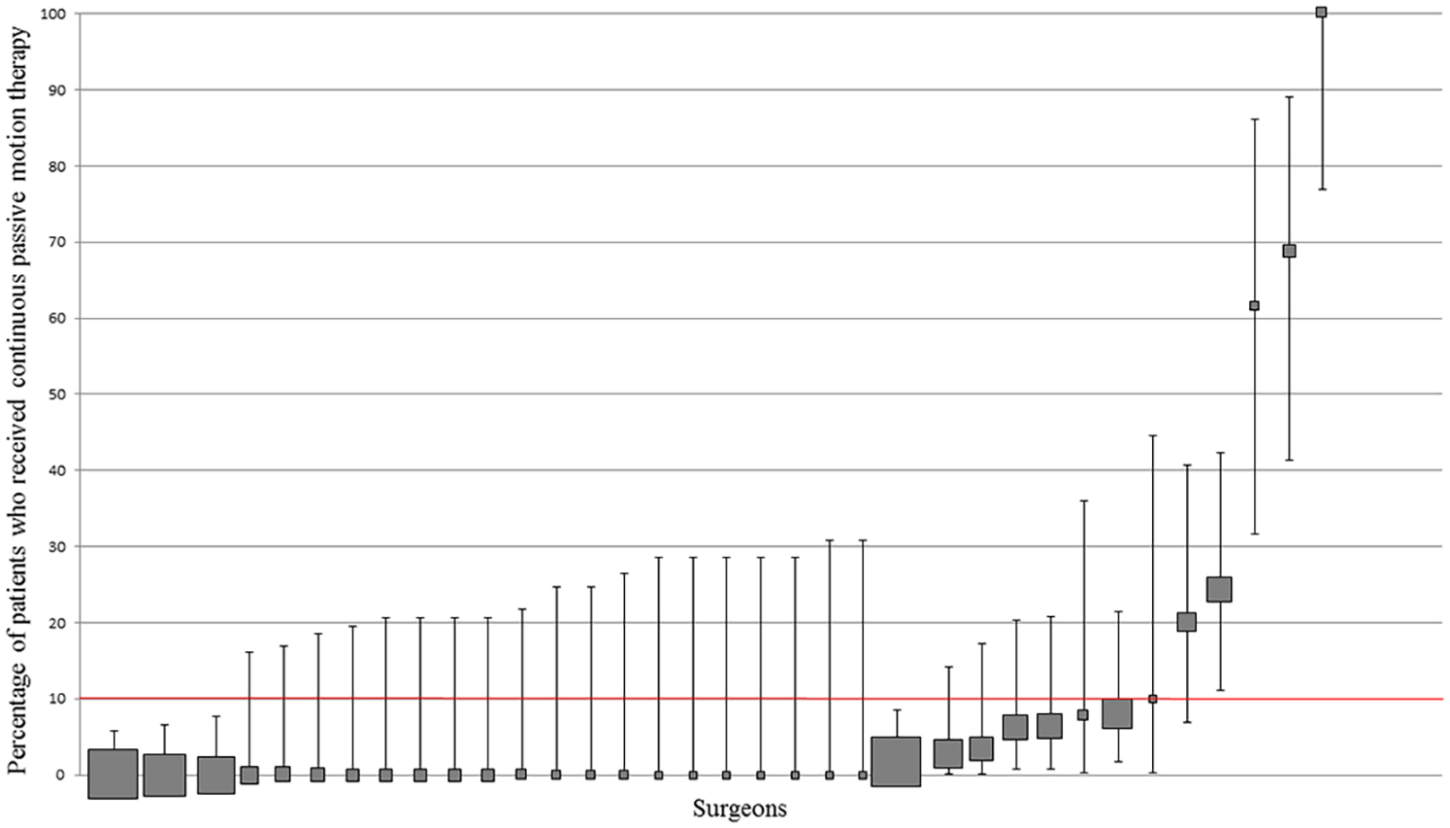
Variation in CPM use at surgeon level. Percentage and 95% CI of patients who received CPM per surgeon. Each filled square represents a single surgeon. The size of the filled square represents the relative number of patients operated on by each surgeon. Surgeons who operated on less than 10 patients were excluded. The red line indicates where utilisation should be based on whether the literature supports routine practice (≥90% use) or does not support routine practice (<10% use).

The frequency with which CPM was used routinely by each surgeon ranged from 0% to 100% (Fig 4). 3% (1/35) of surgeons used CPM ≥ 90% of the time. 85.7% (30/35) of surgeons used it ≤ 10% of the time.

#### Patient-level data

9.1% (96/1059) of patients undergoing TKA received CPM. Removing those who received it due to a specific indication, 69 patients (6.5%) were prescribed CPM routinely. Notably, all 69 patients who received CPM were from four of the 19 hospitals included in the study.

### Tranexamic acid (TXA) (Cyklokapron)

Tranexamic acid (TXA) acts as a competitive inhibitor of plasminogen and thereby delays fibrinolysis [26] and has been reported to reduce perioperative blood loss and the need for allogenic transfusion without an increase in thromboembolic events following orthopaedic surgery [27–29].

#### Recommendations from the literature

16 meta-analyses were found [30–45] (Table B in S1 Appendix). All meta-analyses showed a significant reduction in blood loss and donor transfusion rate when tranexamic acid was applied either topically (via intra-articular injection or as part of joint irrigation) or intravenously during or after TKA or THA. There was no increase in thromboembolic or other adverse effects. In the four meta-analyses comparing intravenous to topical application of TXA, no significant differences in the measured outcomes were reported. The routine use of TXA in knee and hip arthroplasty was supported by all 16 reviews with benefits being greater with doses over 2g/100ml (20mg/ml) [33]. Mean AMSTAR score for the included reviews was 50% (range 36–73%).

#### Surgeon practice—High volume cohort

**Fig 5:**
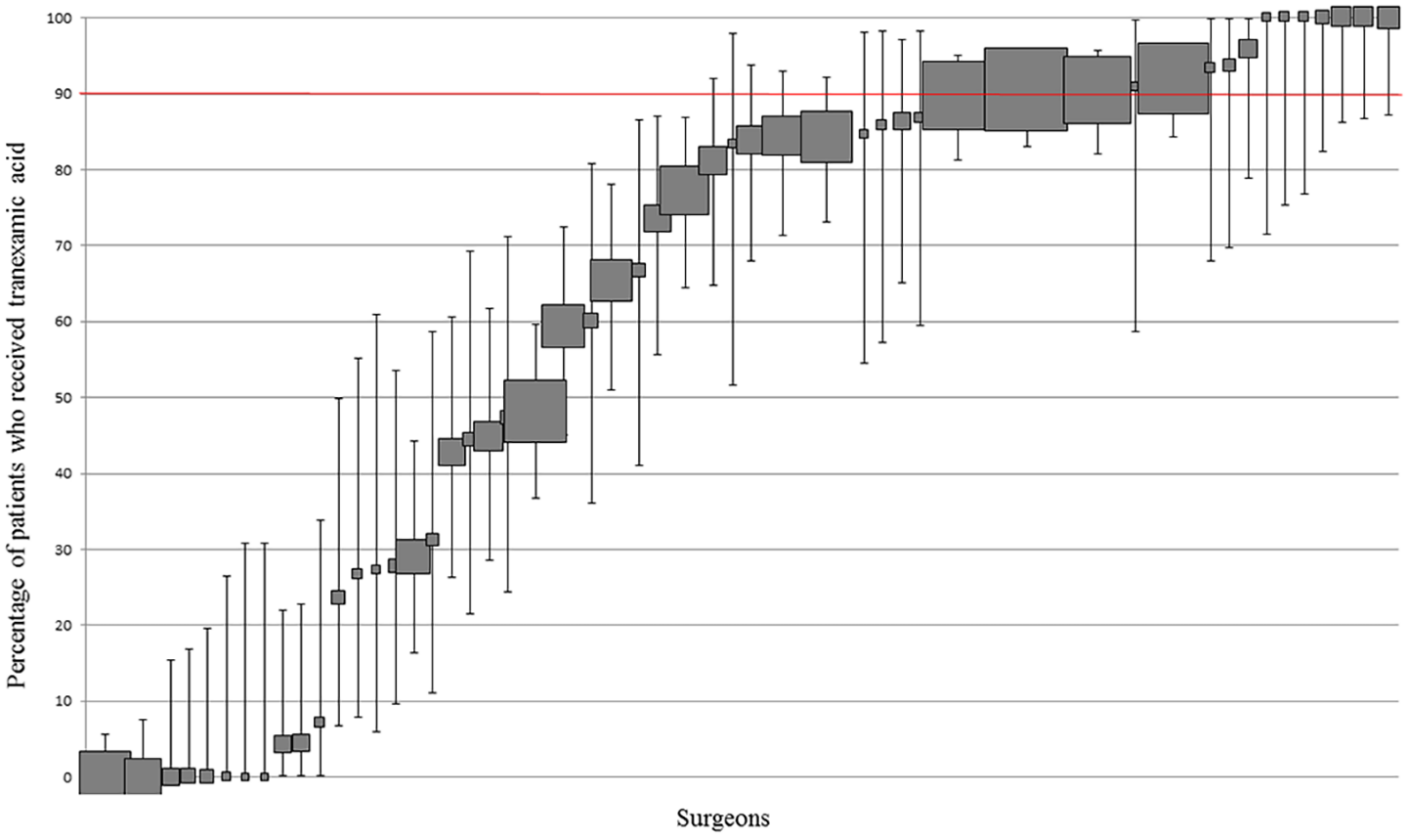
Variation in TXA use at surgeon level. Percentage and 95% CI of patients who received TXA per surgeon. Each filled square represents a single surgeon. The size of the filled square represents the relative number of patients operated on by each surgeon. Surgeons who operated on less than 10 patients were excluded. The red line indicates where utilisation should be based on whether the literature supports routine practice (≥90% use) or does not support routine practice (<10% use).

The frequency of surgeon use ranged from 0 to 100% (Fig 5); 26.9% (14/52) of surgeons used TXA ≥ 90% of the time. 21.2% (11/52) of surgeons used it ≤ 10% of the time.

#### Patient-level data

60.4% (1145/1895) of patients undergoing TKA or THA received TXA. The route of administration was not stated in 82.3% of cases. We undertook a sensitivity analysis to determine if the percentage who received it increased if those with an identifiable potential contra-indication to the use of TXA—history of venous thromboembolism, stroke, vision impairment [46, 47]–were excluded. In the remaining sample, TXA was used in 61.1% (1068/1749) of patients. There were no clear between-site patterns of use.

### Cryotherapy

Cryotherapy, in the context of arthroplasty, is defined as the use of ice bags or cold water covering the surgical site [48], and is used with the aims of decreasing swelling, pain and inflammation in and around the operated joint.

#### Recommendations from the literature

One systematic review (TKA) and three meta-analyses (two TKA, one TKA+THA) were found [16, 48–50] (Table C in S1 Appendix).

TKA: All meta-analyses found that cryotherapy produced a small, statistically significant reduction in postoperative blood loss and a transient reduction in knee pain acutely. Three of the reviews [16, 48, 50], however, concluded that the clinical relevance of these reductions was questionable as transfusion requirements were not lower and there were no longer-term benefits. Thus, routine use is not supported.

THA: One meta-analysis [49] investigated the effect of cryotherapy on blood loss and pain following THA. No differences were observed between those receiving or not receiving cryotherapy on these outcomes except for a transient decrease in hip pain on the second post-operative day. Routine use is not supported. Mean AMSTAR score for the included reviews was 48% (range 0–100%).

#### Surgeon practice—High volume cohort

**Fig 6:**
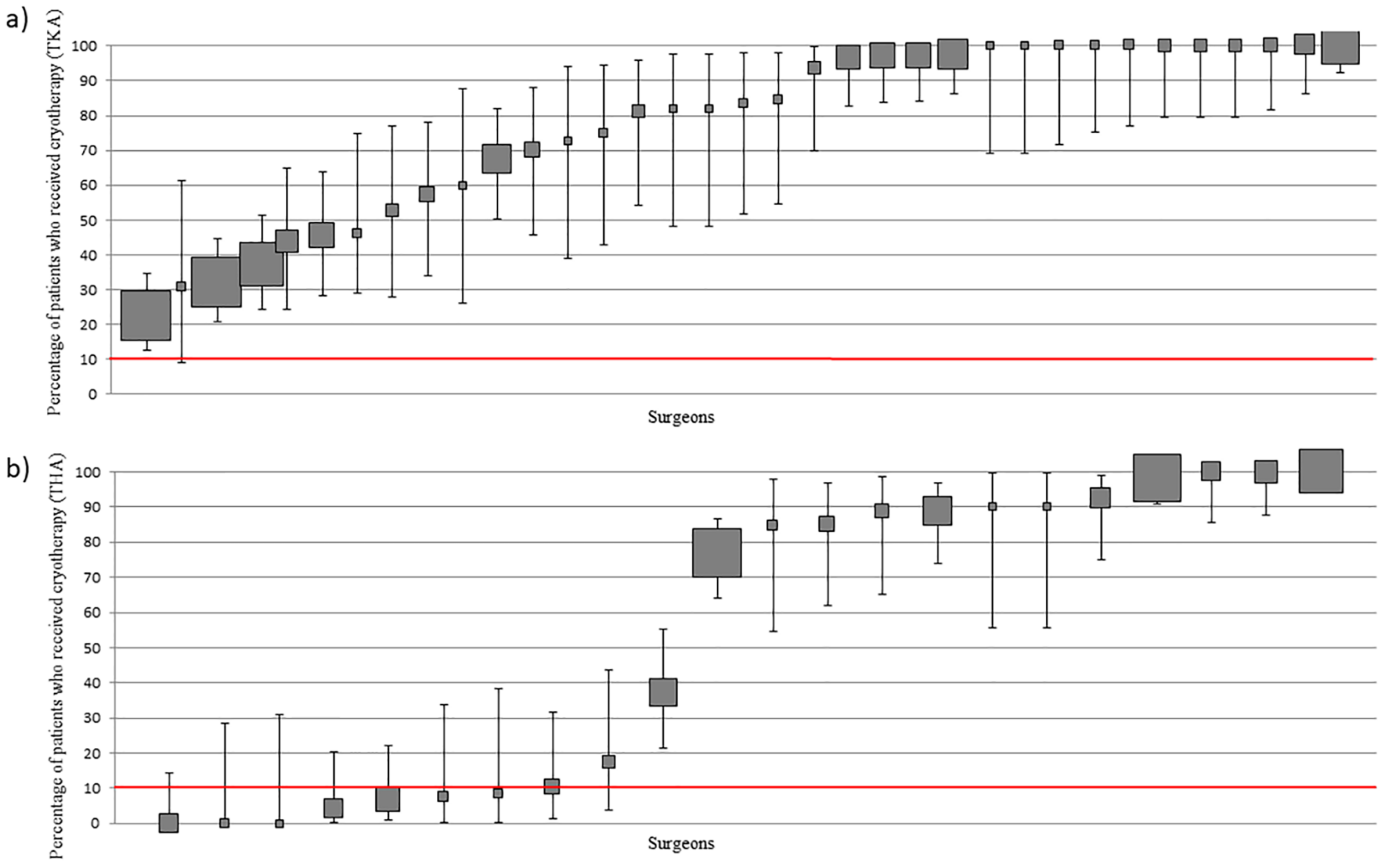
(a): Variation in cryotherapy use at surgeon level (TKA) (b): Variation in cryotherapy use at surgeon level (THA). Percentage and 95% CI of patients who received cryotherapy per surgeon. Each filled square represents a single surgeon. The size of the filled square represents the relative number of patients operated on by each surgeon. Surgeons who operated on less than 10 patients were excluded. The red line indicates where utilisation should be based on whether the literature supports routine practice (≥90% use) or does not support routine practice (<10% use).

The frequency of surgeon use ranged from 22.2% to 100% for TKA and 0% to 100% for THA (Fig 6a and 6b). 45.7% (16/35) of surgeons used cryotherapy ≥ 90% of the time following TKA whilst 0% (0/35) used it ≤ 10% of the time 31.8% (7/22) of surgeons used cryotherapy ≥ 90% of the time following THA and 36.3% (8/22) used it ≤ 10% of the time.

#### Patient-level data

72.4% (773/1068)) of patients undergoing TKA and 54.1% (447/826) undergoing THA received cryotherapy. Cryotherapy was routinely used in 11 out of 19 sites following TKA and in 8 out of 19 sites following THA. It is noteworthy that 8 of 11 sites routinely using cryotherapy following knee arthroplasty and 6 of 8 sites routinely using it following hip arthroplasty, were private. No patient received cryotherapy based on a specific indication.

### Intra-articular drainage

Intra-articular drains inserted intra-operatively are widely used to reduce formation of haematoma in different surgical specialities [51], but they have been questioned in orthopaedic surgery due to the potential for adverse effects such as wound infection and higher need for red blood cell transfusion [52–55].

#### Recommendations from the literature

One systematic review (THA) and seven meta-analyses (five THA, two TKA) were found [56–63] (Table D in S1 Appendix).

TKA: Two meta-analyses [57, 63] showed an increased blood loss and need for blood transfusion with the use of closed-suction intra-articular drainage. Therefore, routine use was not supported.

THA: Six reviews [56, 58–62] including five meta-analyses all showed an increased blood loss as well as an increased need for blood transfusion. Five of these reviews concluded that routine use was not supported, however, one review concluded that drains may be beneficial as their use was associated with less wound-related complications. Mean AMSTAR score for the included reviews was 39% (range 0–64%).

#### Surgeon practice—High volume cohort

**Fig 7:**
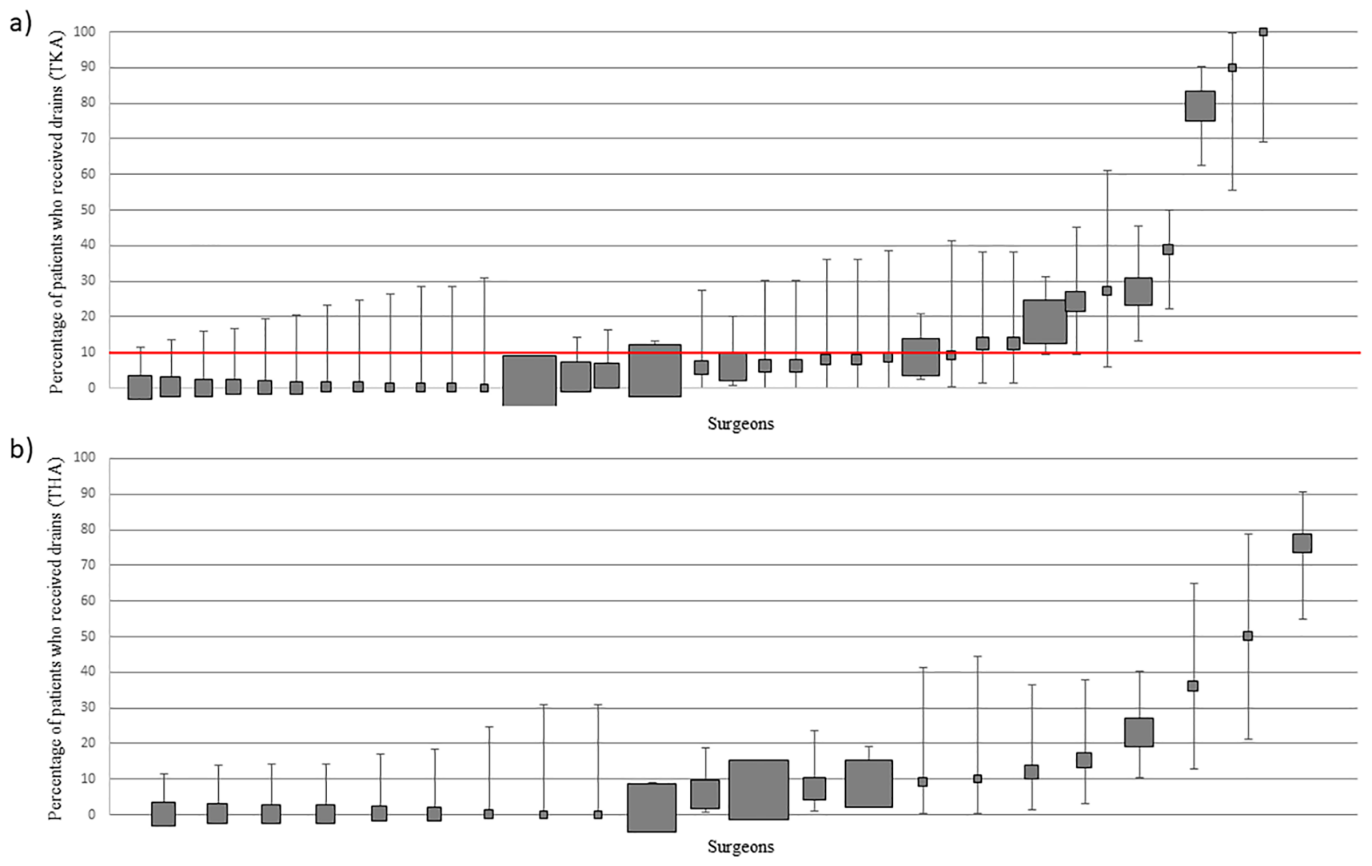
(a): Variation in drainage use at surgeon level (TKA) (b): Variation in drainage use at surgeon level (THA). Percentage and 95% CI of patients who received intra-articular drainage per surgeon. Each filled square represents a single surgeon. The size of the filled square represents the relative number of patients operated on by each surgeon. Surgeons who operated on less than 10 patients were excluded. The red line indicates where utilisation should be based on whether the literature supports routine practice (≥90% use) or does not support routine practice (<10% use).

The frequency of surgeon use ranged from 0% to 100% for TKA and 0% to 76% for THA (Fig 7a and 7b). 5.7% (2/35) of surgeons used drains ≥90% of the time and 71.4% (25/35) used them ≤10% of the time in TKA. 0.0% (0/22) of surgeons used drains ≥90% of the time and 72.7% (16/22) of surgeons used them ≤10% of the time in THA. It is noteworthy that if a surgeon used drains, they used it for both their TKA and THA patients. [Note: Patients who received reinfusion drains alone or prior to a closed suction drain were included in the ‘no drain‘ group because the rationale for utilising the reinfusion drain differs from the rationale for using closed suction or free drainage.]

#### Patient-level data

50% (534/1070) of patients undergoing TKA and 37.0% (302/823) undergoing THA received a surgical drain (closed-suction alone or following removal of a reinfusion drain). Ignoring those who received a re-infusion drain first, closed suction drains were used in 13.3% of knee and 12.5% of hip arthroplasties. There was no overt pattern regarding the use of surgical drains and hospital.

### Antibiotic loaded bone cement (ALBC)

Antibiotic loaded bone cement is used with the primary aim of reducing deep surgical infections following TKA or THA [64]. Its use has been questioned due to uncertainty concerning its capacity to prevent surgical site infection as well as the possibility of adverse effects [65–67].

#### Recommendations from the literature

Three meta-analyses (one primary TKA, two primary TKA+THA) were found [68–70] (Table E in S1 Appendix). The conclusions from the reviews for the use of ALBC in TKA were consistent, whilst they were not for THA.

TKA: Two meta-analyses [68, 69] concluded that the use of ALBC does not decrease the rate of deep or superficial infections compared to plain bone cement. One meta-analysis [70], including both TKA and THA surgeries, found a significantly lower risk of deep infections and no effect on the risk of superficial infections when ALBC was used. A sub-group analyses in this same study found that the reduction in deep infection was only evident in the THA group. Routine use is not supported.

THA: One meta-analysis concluded that ALBC did not lower the rate of deep or superficial infection rate [69]. As described above, another meta-analysis concluded ALBC reduced the rate of deep infection in THA, but not TKA surgeries [70]. No recommendation is made about ALBC for THA. Mean AMSTAR score for the included reviews was 46% (range 36–55%).

#### Surgeon practice—High volume cohort

**Fig 8:**
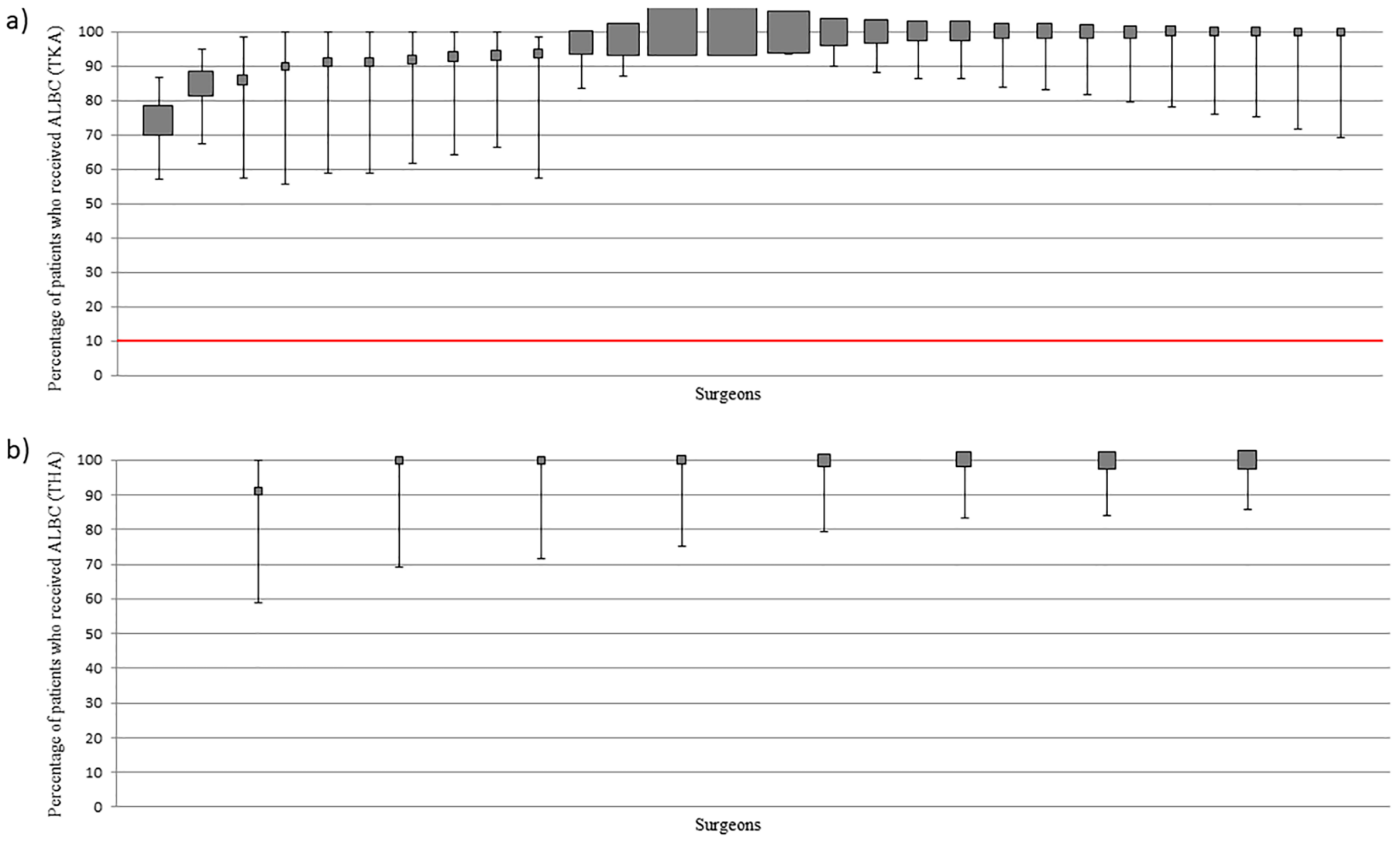
(a): Variation in ALBC use at surgeon level (TKA) (b): Variation in ALBC use at surgeon level (THA). Percentage and 95% CI of patients who received ALBC per surgeon. Each filled square represents a single surgeon. The size of the filled square represents the relative number of patients operated on by each surgeon. Surgeons who operated on less than 10 patients were excluded. The red line indicates where utilisation should be based on whether the literature supports routine practice (≥90% use) or does not support routine practice (<10% use).

In those surgeons who used cement fixation, the frequency of surgeon use ranged from 74 to 100% in TKA and from 91 to 100% for THA (Fig 8a and 8b). 90.0% (26/29) of surgeons used it in ≥90% of cemented fixation for TKA and 0% (0/29) of surgeons used it in ≤10%. 100.0% (8/8) of surgeons used it in ≥90% of cemented fixation for THA and 0% (0/8) of surgeons used it in ≤10%.

#### Patient-level data

Where cement fixation of prostheses (some or all components) was used, ALBC was used in 96.0% (910/948) of patients for TKA and 95.4% (247/259) of patients for THA. There was no clear pattern of use across hospitals.

### Patella resurfacing

Resurfacing the patella was introduced to reduce the rate of anterior knee pain (AKP) following TKA [71–73]. However, the resurfacing itself is associated with complications such as patella fracture, instability, avascular necrosis and patellar tendon injury [74, 75].

#### Recommendations from the literature

Two systematic reviews and nine meta-analyses were found [22, 76–85] (Table F in S1 Appendix). All reviews concluded that patella resurfacing (compared to non-resurfacing) results in a lower risk of reoperation and no differences concerning patient-rated function and satisfaction, but they vary in terms of the effect of resurfacing on anterior knee pain. One review [80] showed a reduction in AKP while seven [76, 78, 79, 81–84] found no significant decrease if the patella was resurfaced. In conclusion, there are inconsistent recommendations for routinely resurfacing or not resurfacing the patella in TKA. Resurfacing may be justified by the reduction in revision surgery, but the opposite may also be argued given the uncertain benefits to knee pain, function, and satisfaction and adverse events associated with resurfacing at the time of the primary surgery. Mean AMSTAR score for the included reviews was 29% (range 0–55%).

#### Surgeon practice—High volume cohort

**Fig 9:**
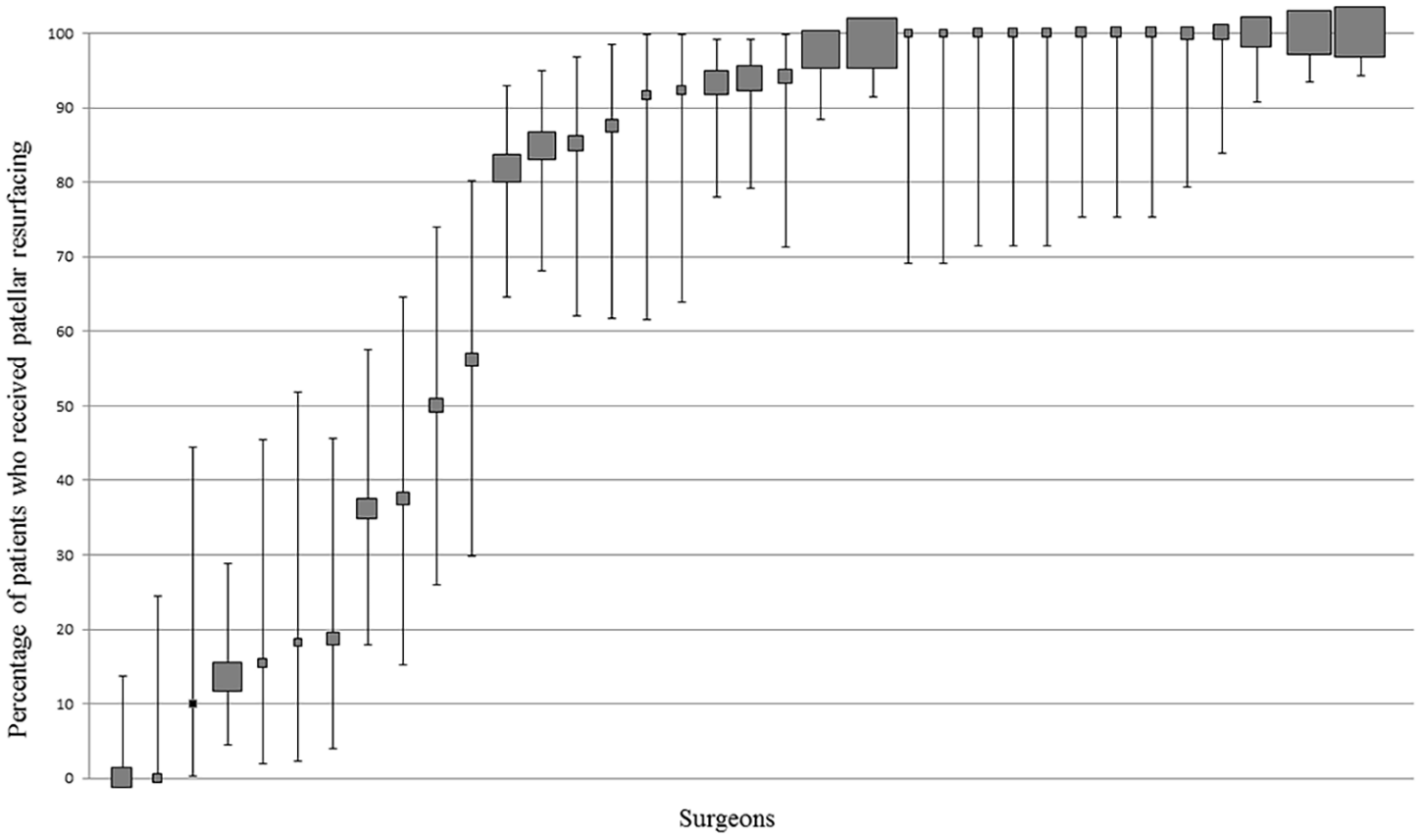
Variation in patellar resurfacing at surgeon level. Percentage and 95% CI of patients with patellar resurfacing per surgeon. Each filled square represents a single surgeon. The size of the filled square represents the relative number of patients operated on by each surgeon. Surgeons who operated on less than 10 patients were excluded. The red line indicates where utilisation should be based on whether the literature supports routine practice (≥90% use) or does not support routine practice (<10% use).

The frequency of surgeon use ranged from 0 to 100% (Fig 9); 57.1% (20/35) of surgeons resurfaced the patella ≥90% of the time and 8.6% (3/35) resurfaced it ≤10% of the time.

#### Patient-level data

74.0% (786/1065) of patients undergoing TKA received patella resurfacing. No between-hospital patterns of use were found.

### Indwelling urinary catheters

Urinary catheters are inserted prior to surgery with the aim of preventing post-operative urinary retention (POUR). The reported incidence of POUR varies widely—from 0 to 70%—following TKA or THA [86].

#### Recommendations from the literature

One meta-analysis was found [87] (Table G in S1 Appendix). The meta-analysis showed a lower risk of POUR following catheterization with removal within 24–48 hours and no increase in urinary tract infection compared to intermittent catheterization. Therefore, the routine use of IDCs for TKA or THA would appear to be reasonable. AMSTAR score for the included review was 73%.

#### Surgeon practice—High volume cohort

**Fig 10:**
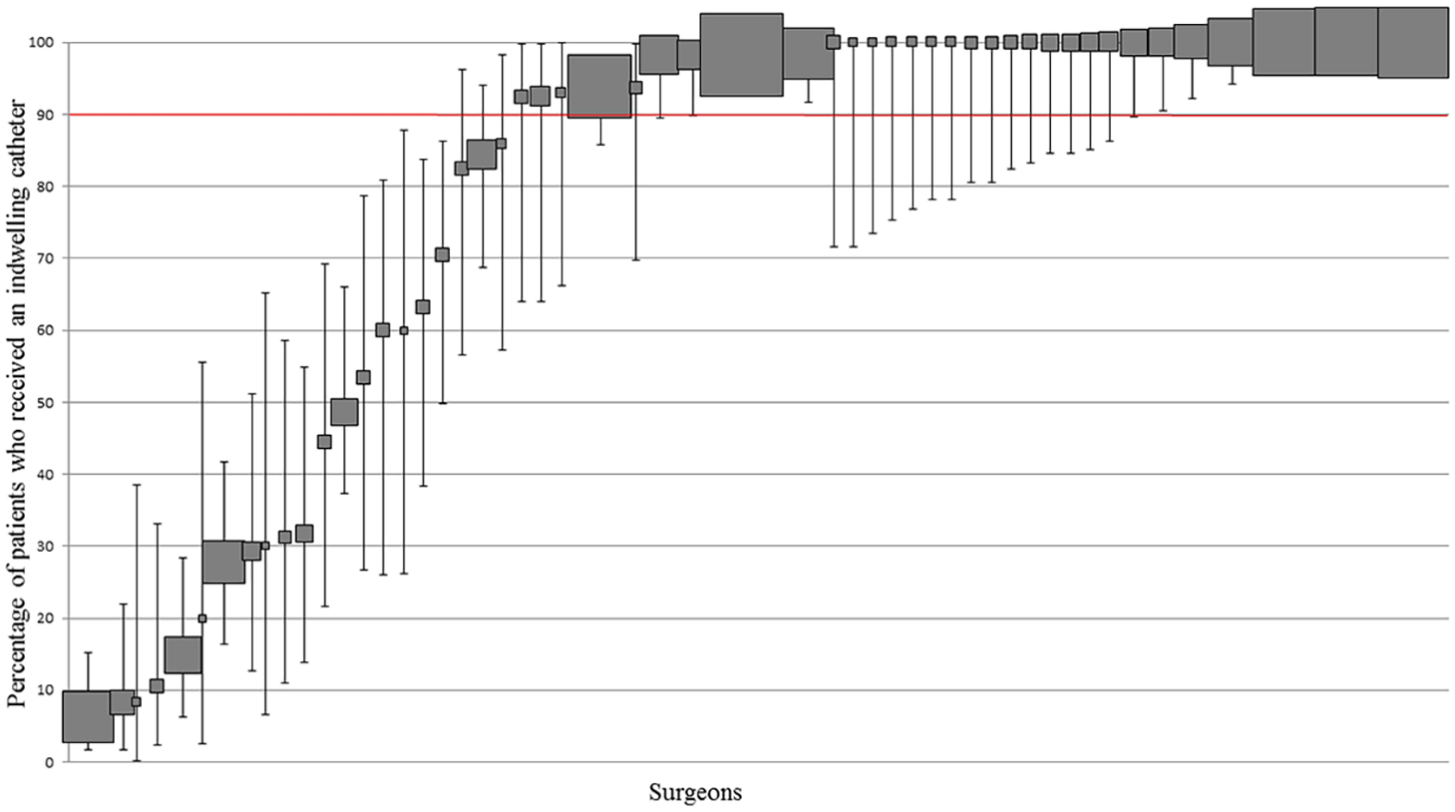
Variation in IDC use at surgeon level. Percentage and 95% CI of patients who received IDC per surgeon. Each filled square represents a single surgeon. The size of the filled square represents the relative number of patients operated on by each surgeon. Surgeons who operated on less than 10 patients were excluded. The red line indicates where utilisation should be based on whether the literature supports routine practice (≥90% use) or does not support routine practice (<10% use).

The frequency of surgeon use ranged from 6 to 100% (Fig 10); 59.6% (31/52) of surgeons used it ≥ 90% of the time. 5.8% (3/52) of surgeons used it ≤ 10% of the time.

#### Patient-level data

78.3% (1486/1898) of patients undergoing TKA or THA received an IDC inserted peri-operatively. In 9 of 19 sites (public and private), IDCs were used routinely.

#### Surgeon and hospital variation based on ICCs

Table 2 summarises the ICCs based on the 3-level multilevel models. The model for THA cement was not able to be estimated since all patients used in analysis received the intervention. There was significant surgeon-level variation in each intervention with ICCs ranging between 0.28 and 0.82. Of these, hospital significantly explained surgeon-level variation for four interventions. For use of IDCs, tranexamic acid, and CPM, 21%, 11%, and 48% of the between-surgeon variation respectively, was significantly explained by the hospital. For the use of cryotherapy in TKA, 100% of the between-surgeon variation was significantly explained by 'hospital'.

**Table 2:** Mixed modelling analyses.

	IDC	TXA	TKA cement	THA cement	TKA CPM	TKA Cryotherapy	THA Cryotherapy	TKA Patella	TKA drains	THA drains
ICC Surgeon relative to Patients	0.8*	0.74*	0.28*	NA	0.81*	0.64*	0.78*	0.82*	0.63*	0.49*
ICC Hospital relative to Surgeon	0.21^#^	0.11^##^	0	NA	0.48*	1*	0	0	0	1

ICC, intra-class correlation; IDC, indwelling catheter; TXA, tranexamic acid; TKA, total knee arthroplasty; THA, total hip arthroplasty; CPM, continuous passive motion;

*p < 0.001;

^#^p = 0.01;

^##^p = 0.02.

NA, All patients had antibiotic cement therefore model not able to be estimated.

Interpretation examples: For IDC, total proportion of variance that is between surgeons is 80% (ICC surgeon = 0.8). Of that 80%, 21% (ICC hospital = 0.21, p = 0.01) of variance is between hospitals. For TKA cement use, total proportion of variance that is between surgeons is 28%, and none of that is due to differences between hospitals (ICC hospital = 0, p = 1). For TKA cryotherapy use, 64% of the total variation is between surgeons, however, all of that is attributable to differences between hospitals (ICC hospital = 1, p < 0.0001).

## Discussion

Variation in care practices is thought to contribute to the wide variation in costs seen for arthroplasty surgery [88–92], but no studies have documented specifically what the variations in acute care practices are at the surgeon level. This study provides a cross-sectional analysis of surgeon level variation across a range of interventions currently or historically relevant to TKA or THA.

High level evidence should be a strong driver for determining clinician practice so that best outcomes are achieved for the majority [93]. Given this, we could expect no or small variation in practice when evidence is consistent, and wide variation when there is a lack of consistency for or against an intervention. In support of the latter, in other settings such as in primary care, wide variation has been observed for interventions or processes when discretionary clinical decision-making is required; that is, when the evidence is lacking or inconclusive [94].

Of the seven interventions examined here, routine use is supported by the high-level evidence for two only (TXA and indwelling catheterisation) and non-routine use is supported for four (CPM, cryotherapy, intra-articular drainage (TKA), ABLC (TKA)). No recommendations for routine use or otherwise are made for three interventions—the use of ABLC and intra-articular drains for THA, and patella resurfacing.

It should be noted that although our recommendations were based on a high level of evidence (SRs), compliance with the AMSTAR tool was only moderate. This suggests that there is room for improvement for the methodological quality of the available evidence. However, the available SRs remain the strongest study design to base our recommendations on [95].

For the two interventions which are recommended to be used routinely, the frequency of routine use by surgeon was not particularly high with less than 60% of surgeons using IDCs routinely and even fewer (27%) using TXA routinely.

For the interventions which are not recommended to be used routinely, surgeon practice was most consistent with the evidence for CPM—with almost 86% of surgeons using CPM ≤ 10% of the time—and intra-articular closed suction drains (TKA)—with 71.4% of surgeons using them ≤ 10% of the time. For the remaining interventions, non-routine use was significantly greater than routine use for intra-articular drainage (THA) and marginally greater for cryotherapy (THA). Routine use was far greater than non-routine use for cryotherapy (TKA) and ABLC (TKA and THA). Though no clear recommendations are available regarding patella resurfacing, routine use was more frequent than non-routine use.

Our observations indicate that even in the presence of consistent high-level evidence, between-surgeon practice variation is present. It is unclear what is driving the variation we observed as our study did not seek to determine the cause of any variation found. It is possible for all the interventions examined that lack of awareness of the evidence is a driver. For example, a change in recommendation for the use of ABLC for TKA has been made over recent years. Whilst most literature published before 2010 favoured ALBC use for TKA as it was reportedly associated with a lower infection rate [96, 97], the more recent high-level studies (published from 2010) do not confirm this finding [96, 97].

It is also possible that surgeons choose not to follow the evidence, and may rely more on their own experiences. For example, anecdotally, many patients believe cryotherapy is of benefit. Consequently, surgeons may choose to align their views with those who have experienced the intervention rather than with evidence that may not have captured the patient perspective. Certainly, a deficit of the cryotherapy evidence is a relative lack of patient-reported benefit with the exception of pain relief measures.

Patient factors may also affect the use of a particular intervention, for example, the presence of a specific indication or contraindication. We allowed for specific indications to drive the application of CPM and cryotherapy. Interestingly, occasions of use for either were rarely if ever justified on the grounds of a specific indication. Regarding the presence of a contraindication, our sensitivity analysis for TXA which removed those with possible contraindications to TXA, yielded similar results. We contend then that given some surgeons used an intervention 100% of the time and others 0% of the time, the presence of a specific indication or contraindication was not a predominant determinant of who received treatment and thus was not a predominant driver of the practice variation observed. This is not to say that patient factors are not relevant and should not influence care. In the case of IDCs, for example, it is possible that the surgeons who did not use IDCs routinely reserved their use for high risk patients. The latter has been recommended previously [87] and is predicated on the idea that the risks and costs of IDCs render it worthwhile to only routinely use them in those most at risk for urinary retention. We did not have sufficient information to determine if use of IDCs for some surgeons was selective based on patient risk. A similar argument may explain the frequency of patella resurfacing; some surgeons may use it some of the time based on the presence of patella-femoral arthritis [98], hence we see many surgeons using it more than 10% but less than 90% of the time.

The multilevel model analyses indicated that for some of the care processes (i.e. IDC, TXA, CPM (TKA) and cryotherapy (TKA)), 'hospital' significantly explained some of the between-surgeon variation. In other words, for some interventions, surgeons within the same hospital behave or practice similarly. This observation is not unexpected and likely explained by the presence of a specific protocol for that process. In this light, what is perhaps surprising then is that hospital was not a significant influence for all practices and only explained a small degree of the between-surgeon variation. This would suggest that consistent protocols within a hospital for all surgeons is not common.

### Strengths and limitations

Our study has several strengths and limitations. Our literature search spanned various databases with no restrictions except the requirement for only the most recent evidence (since 2010). Multiple researchers independently extracted the recommendations and conclusions from each identified source, and group discussion aided the synthesis of recommendations from sources which often conflicted with each other.

Other strengths of our study lie in the size and representativeness of our patient and surgeon cohorts. The patient profile typifies that of arthroplasty cohorts based on age, gender, body mass index and baseline joint impairment as per the Oxford Knee or Hip Scores [2–4, 99]. Our surgeon cohort represents those from the public and private sectors from high-volume centres, thus, the observations here around surgeon-level care are arguably generalizable to the broader orthopaedic surgeon community operating in high-volume centres. We acknowledge, however, that the inclusion of surgeons who contributed low numbers of patients to our cohort—consistent with their low volume of surgery at that particular hospital—contributed to the very wide between-surgeon variation observed. Importantly though, even when we restricted our analysis to those contributing ten or more patients, the range of variation was similar, indicating variable surgeon-level sample sizes do not in themselves explain the wide between-surgeon variation. Another strength is that we provide a summary of the quality of the reporting of the SRs included. Thus, the reader can appreciate whether the reviews overall are well reported. It should be noted that we did not exclude reviews based on an arbitrary AMSTAR score because a low score for a review does not mean the RCT evidence upon which the review is based is poor. It is also worthwhile noting that despite the low AMSTAR scores, there was consistency in the recommendations across SRs in the majority of cases and where they were not consistent, we have reported this.

In terms of study limitations, we have not reported surgeon-level data based on surgeon characteristics so we do not know if surgeon experience (years practised) or annual volume of arthroplasty surgery influences whether surgeon practice aligns with the high-level evidence. The patient-level data reported here is influenced more by the surgeons contributing the most patients to the dataset. We do not know if these contributions reflect their contribution to the annual total volume of TKA or THA surgery in Australia, thus, patient-level variation in care may be over or under-stated here. Finally, this paper does not include all acute-care practices relevant to TKA or THA—such as use of reinfusion drains and spinal anaesthesia—as it was not practicable to include all in one publication.

## Conclusion

In summary, our study summarises the high-level evidence available for several interventions historically of importance to the TKA and THA fields. In doing so, it identifies where deficits or inconsistencies in evidence exist. Further, our study illustrates widespread variation in clinical practice even when consistent high-level evidence is available. In light of the latter, it is unclear whether more evidence would lessen practice variation. If we are to address practice variation in this area, we first must understand the major barriers to the adoption of recommended practices including the clinical reasoning behind discretionary decision-making.

## Supporting information

S1 Appendix (DOCX)
