# Supplementary material for: Evidence base and practice variation in acute care processes for knee and hip arthroplasty surgeries
Source: PLoS One. 2017 Jul 19;12(7):e0180090. doi: 10.1371/journal.pone.0180090 (PMC5516983; doi:10.1371/journal.pone.0180090)
Supplement: S1 Appendix — (DOCX) [file pone.0180090.s001.docx]

**Appendix**

**EVIDENCE BASE AND VARIATION IN ACUTE CARE PROCESSES FOR KNEE AND HIP ARTHROPLASTY SURGERIES**

### Table of contents

[Continuous passive motion - Rationale 2](#_Toc452871230)

[*Table A – Continuous passive motion in TKA* 3](#_Toc452871231)

[Tranexamic Acid (TXA) (Cyklokapron) – Rationale 5](#_Toc452871232)

[*Table B Tranexamic Acid (TXA) in TKA and THA* 6](#_Toc452871233)

[Cryotherapy – Rationale 9](#_Toc452871234)

[*Table C – Cryotherapy in TKA and THA* 10](#_Toc452871235)

[Intraarticular Drains – Rationale 13](#_Toc452871236)

[*Table D Intraarticular drains in TKA and THA* 14](#_Toc452871237)

[Antibiotic loaded bone cement (ALBC) – Rationale 17](#_Toc452871238)

[*Table E Antibiotic cement in TKA and THA* 20](#_Toc452871239)

[Patellar resurfacing – Rationale 21](#_Toc452871242)

[*Table F Patellar resurfacing in TKA* 23](#_Toc452871243)

[Indwelling Urinary Catheters – Rationale 25](#_Toc452871244)

[*Table G Indwelling Catheter in TKA and THA* 27](#_Toc452871245)

[*Figure K – Literature Search Example* 28](#_Toc452871246)

[References 29](#_Toc452871247)

# Continuous passive motion - Rationale

A minimum of 65° knee flexion is needed for walking, 90° for climbing stairs and 105° for standing up from a chair.[1, 2] Most functional activities require a minimum of 103° knee joint excursion.[3] Therefore, a concern after TKA is loss of knee joint range (flexion and/or extension), secondary to intra-articular stiffness. About 1.3% of patients demonstrate a flexion contracture (loss of terminal extension) of ≥15° and/or manifest an end-range flexion of <75° three years after TKA.[4] Significant stiffness may result in a delay in discharge from hospital after the initial surgery, the need for manipulation under anesthesia (MUA) at a later date, or worse longer-term patient reported pain, function or quality of life.[5]

Mobilization started shortly after surgery has shown the potential to reduce the risk of joint stiffness, capsular contractures and to improve cartilage nutrition in rabbit as well as in human knees [6].

A continuous passive motion (CPM) machine is a motorized devise which passively moves the patient’s leg in a predefined range of motion.[7] R. Salter was the first who showed that early continuous passive motion has a positive effect on rehabilitation in rabbit knees.[8] In the 1980s it gained acceptance and was integrated into clinical practice.[6]

Today, early active mobilization, including getting out of bed soon after surgery and actively moving the joint, is standard care following knee arthroplasty.[9] Given the cost of the CPM device and the requirement for the patient to stay in bed, the value of CPM has been questioned.

Table A summarises the conclusions from related systematic reviews and meta-analyses.

Figure A shows variation in percentage of patients who received CPM following TKA and THA per surgeon.

## *Table A – Continuous passive motion therapy in TKA (compared to standard physiotherapy if not specified)*

| Study | Year of publication | Study type | Conclusion |
| --- | --- | --- | --- |
| APTA (American Physiotherapy Association)[10] | 2015 | Guideline | - no significant evidence for improvement of short- or long-term knee extension, long-term knee flexion, long-term function, pain and quality of life - CPM following TKA not recommended for routine use |
| Sanchez et al.[11] | 2015 | Systematic review –  12 clinical trials | - no significant evidence for effects on range of   Joint (long-term), pain, balance, motion, healing and hospital stay by CPM   - improvement of range of joint (flexion) in short-term |
| Nikolaou et al.[12] | 2014 | Systematic review – three meta-analyses (including [13] and [1]) and seven RCTs **(TKA)** | - no evidence of high quality that prefers CPM over other techniques of mobilization - no routine use of CPM recommended |
| Harvey et al.[14] | 2014 | Meta-analysis  (Cochrane Library) –  24 RCTs **(TKA)** | - no clinically significant evidence for improvement of concerning active knee flexion, ROM, pain, function and quality of life - low quality evidence for decrease of manipulation under anesthesia (MUA) and reduction of occurrence of adverse effects - no justification for clinical use in routine |
| Yip et al.[15] | 2010 | Meta-analysis –  five RCTs **(TKA)** | - no clinically significant increase in ROM after use of CPM |
| Harvey et al.[13] | 2010 | Meta-analysis –  20 RCTs  (Cochrane review) **(TKA)** | - clinically insignificant increase of ROM (flexion) and active knee flexion - no effect on LOS and need for manipulation under anesthesia - no justification for clinical use in routine |

Fig A. Variation in continuous passive motion therapy use at surgeon level

Percentage of patients who received continuous passive motion

therapy

Surgeons

Percentage and 95% CI of patients who received CPM per surgeon. Each filled square represents a single surgeon. The size of the filled square represents the relative number of patients operated on by each surgeon. Surgeons who operated on less than 2 patients were excluded.

# Tranexamic Acid (TXA) (Cyklokapron) – Rationale

Intra- and postoperative blood losses are reportedly up to 1500-1600 ml following TKA (24) or THA (25). Consequently, allogenic transfusion of red blood cells may be necessary. As allogenic transfusion itself is associated with risk, interventions that prevent blood loss in the first place are preferred.

Tranexamic acid (TXA) is a synthetic derivate of the amino acid lysine and acts as a competitive inhibitor of plasminogen thereby delaying fibrinolysis.[16] TXA is routinely used in other surgical fields, including cardiac, dental and prostate surgeries.[17-19] Several studies report a positive effect (decrease) on perioperative blood loss and allogenic transfusion without an increase in thromboembolic events in orthopedic surgery [20-22].

Table B summarises the conclusions from related systematic reviews and meta-analyses.

Figure B shows variation in percentage of patients who received TXA following TKA and THA per surgeon.

## *Table B - Tranexamic Acid (TXA) in TKA and THA (compared to no TXA, if not specified)*

| Study | Year of publication | Study type | Conclusion | |  |
| --- | --- | --- | --- | --- | --- |
| Chen et al.[23] | 2016 | Meta-analysis –  14 RCTs and cohort studies **(THA; topical use of TXA)** | - significant reduction of blood loss, drainage loss and drop of hemoglobin (Hb) level - no significant influence on LOS, risk of deep vein thrombosis (DVT) and pulmonary embolism (PE) |  |  |
| Wei et al.[24] | 2015 | Meta-analysis –  39 RCTs **(TKA; topical, intravenous (IV), oral, intraarticular (IA) use of TXA)** | - significant reduction of blood loss and need for allogenic blood products - no significant influence on risk of DVT or PE |  |  |
| Xu et al.[25] | 2015 | Meta-analysis –  four RCTs **(THA; topical use of TXA)** | - significant reduction of blood loss and transfusion rate - no significant influence on risk of thromboembolic events |  |  |
| Yue et al.[26] | 2015 | Meta-analysis –  12 trials **(TKA; topical use of TXA)** | - significant reduction of blood loss, need for transfusion, postoperative drain output and Hb drop - higher concentration of TXA (over 2g/100ml) with better outcome than lower concentration - no significant influence on risk of DVT or PE - concentrated topical use of TXA should be used in TKA |  |  |
| Wu et al.[27] | 2015 | Meta-analysis –  34 RCTs **(TKA; intravenous, intraarticular use of TXA)** | - significant reduction of total blood loss, postoperative blood loss, transfusions/patient and postoperative drop of Hb when both IV or IA used - no reduction in terms of intraoperative blood loss - no significant influence on adverse events |  |  |
| Shemshaki et al.[28] | 2015 | Meta-analysis –  31 RCTs **(TKA; IV compared to IA use of TXA)** | - significant reduction of total blood loss and transfusion rate - no significant differences between IA and IV use of TXA |  |  |
| Wang et al.[29] | 2015 | Meta-analysis –  four RCTs and four non-RCTs **(TKA; topical compared to IV use of TXA)** | - no significant differences concerning loss of blood, rate of transfusion or adverse effects between IV and topical use of TXA |  |  |
| Zhang et al.[30] | 2014 | Meta-analysis –  seven RCTs **(TKA; IA use of TXA)** | - significant reduction of blood loss, rate of transfusion and drop of Hb - no significant influence on risk of DVT or PE - TXA as an effective and safe method in TKA |  |  |
| Zhao-Yu et al.[31] | 2014 | Meta-analysis –  six RCTs **(TKA; IA use of TXA)** | - significant reduction of blood loss and need for transfusion - no significant influence on adverse events |  |  |
| Wang et al.[32] | 2014 | Meta-analysis –  six RCTs **(TKA; topical compared to IV use of TXA)** | - no significant differences concerning loss of blood, rate of transfusion or adverse effects between IV and topical use of TXA) |  |  |
| Alshryda et al.[33] | 2014 | Meta-analysis –  14 RCTs **(THA and TKA; topical use of TXA)** | - significant reduction of need for blood transfusion in both TKA and THA - no significant increase of thromboembolic events |  |  |
| Panteli et al.[34] | 2013 | Meta-analysis –  seven studies **(TKA; topical use of TXA)** | - significant reduction of blood loss, need of transfusion and post-operative drainage output - no significant increase of thromboembolic effects |  |  |
| Gandghi et al.[35] | 2013 | Meta-analysis –  33 RCTs **(TKA and THA; topical use of TXA)** | - significant decrease of blood loss and need for allogenic transfusion products in both TKA and THA - no significant increase of DVT | | |
| Fu et al.[36] | 2013 | Meta-analysis –  22 RCTs **(TKA; IV use of TXA)** | - significant decrease of blood loss, rate of transfusion and volume of transfusion - no significant increase of thromboembolic events |  |  |
| Zhang et al.[37] | 2012 | Meta-analysis –  15 RCTS **(TKA; IV use of TXA)** | - ­significant reduction of blood loss (intra- and postoperative) and lower rate of patients needing blood transfusion - no significant increase of risk of adverse effects |  |  |
| Sukeik et al.[38] | 2011 | Meta-analysis –  11 RCTS **(THA; IV use of TXA)** | - significant reduction of blood loss (intra- and postoperative) and proportion of patients needing blood transfusion - no significant increase of thromboembolic events or infections |  |  |

Fig B. Variation in tranexamic acid use at surgeon level

Surgeons

Percentage of patients who received tranexamic acid

Percentage and 95% CI of patients who received TXA per surgeon. Each filled square represents a single surgeon. The size of the filled square represents the relative number of patients operated on by each surgeon. Surgeons who operated on less than two patients were excluded.

# Cryotherapy – Rationale

Cryotherapy in the context of arthroplasty is defined as the use of ice bags or cold water covering the surgical site.[39] It is used with the aims of decreasing swelling, pain and inflammation in and around the operated joint. Although there are reports of adverse effects like infection, necrosis or clots after application of cryotherapy, overall it is a relatively safe postoperative care intervention.[40, 41] In evaluating the efficacy of cryotherapy, the cost of the intervention should also be considered.

Table C summarises the conclusions from related systematic reviews and meta-analyses.

Figure C shows variation in patients receiving cryotherapy following TKA per surgeon.

Figure D shows variation in percentage of patients who received cryotherapy following THA per surgeon.

## *Table C – Cryotherapy in TKA and THA (compared to no cryotherapy if not specified in table)*

| Study | Year of publication | Study type | Conclusion |
| --- | --- | --- | --- |
| Ni et al.[42] | 2015 | Meta-analysis –  12 RCTs **(THA + TKA)** | - significant decrease of pain at second postoperative day - statistically significant decrease of blood loss in TKA - no significant decrease of blood loss in THA - no significant decrease of pain at first and third   postoperative day   - no adverse effects |
| Adie et al.[43] | 2012 | Meta-analysis – 11 RCTs and one controlled clinical trial **(TKA)** | - too small evidence for reduction of blood loss,   reduction of postoperative pain and increase of ROM  justify routine use |
| Markert et al.[44] | 2011 | Systematic review – one meta-analysis[43], and seven studies **(TKA)**; two studies in arthroscopy; one study in ACL reconstruction | - no significant evidence for improvement of ROM by cold compression - decrease of pain by cold compression - decrease in swelling and blood loss by cold compression - cost aspect not considered - possibility of no clinically significant efficacy of cryotherapy |
| Adie et al.[39] | 2010 | Meta-analysis – 11 RCTs **(TKA)** | - significant reduction of blood loss - significant reduction of pain at second postoperative day (without need of more analgesia) - no significant increase of ROM - no benefit concerning swelling - uncertain clinical significance of cryotherapy |

Fig C. Variation in cryotherapy use at surgeon level (TKA)

Percentage of patients who received cryotherapy (THA)

Surgeons

Percentage and 95% CI of patients who received cryotherapy per surgeon. Each filled square represents a single surgeon. The size of the filled square represents the relative number of patients operated on by each surgeon. Surgeons who operated on less than two patients were excluded.

Fig D. Variation in cryotherapy use at surgeon level (THA)

Surgeons

Percentage of patients who received cryotherapy (THA)

Percentage and 95% CI of patients who received cryotherapy per surgeon. Each filled square represents a single surgeon. The size of the filled square represents the relative number of patients operated on by each surgeon. Surgeons who operated on less than two patients were excluded.

# Intraarticular Drains – Rationale

Formation of haematoma follows each surgery. Following splenectomy, nephrectomy and renal transplantation, haematoma formation increases the risk of infection, wound dehiscence and decreases perfusion[45]. A strategy to diminish haematoma is the use of a surgical drain inserted intraoperatively with low pressure connected via perforated plastic tubes. The drain, theoretically, decreases the postoperative collection of blood in the closed wound. Drains

In the 1960s, Waugh was the first to show a potentially positive effect of surgical drains on the postoperative infection rate in orthopedic surgery[46]. Following this, drains were widely used for TKA[47] and THA surgeries[48]. Recent studies have questioned the use of intra-articular drains, however, as they have been shown to increase blood loss, increase the need for donor blood, and increase the rate of infection [49-52].

Table D summarises the conclusions from related systematic reviews and meta-analyses

Figure E shows variation in percentage of patients who received an intraarticular drain following TKA per surgeon.

Figure F shows variation in percentage of patients who received an intraarticular drain following THA per surgeon.

## *Table D - Intraarticular drains in TKA and THA (compared to no drains if not specified)*

| Study | Year of publication | Study Type | Conclusion | |
| --- | --- | --- | --- | --- |
| Zan et al.[53] | 2016 | Meta-analysis –  12 RCTSs **(THA)** | - significant evidence for increase of LOS, operation time, rate of patients needing blood transfusion and units of transfusion per patient - significant reduction of wound-related complications, patients needing dressing reinforcement and hematoma and decreased change in mid-thigh circumference - no effects on blood loss, volume of hematoma or thromboembolic events |  |
| Quinn et al.[47] | 2015 | Meta-analysis –  six RCTs **(TKA)** | - no significant improvement of ROM or decrease of postoperative Hemoglobin drop - no significant differences in circumference of knee - no support of routine use in primary TKA |  |
| Kelly et al.[54] | 2014 | Meta-analysis –  16 studies **(THA)** | - significantly higher loss of blood, LOS and need for blood transfusion - significantly lower post-operative swelling - no significant difference in postoperative pain - routine use in THA not supported |  |
| Chen et al.[48] | 2014 | Meta-analysis –  16 randomized or quasi-randomized controlled trials **(THA)** | - significantly increased need for blood transfusion products and persistent discharge - no clinically significant effects on occurrence of hematoma, dehiscence, DVT, ROM or wound infections - no support of routine use in THA |  |
| Zhou et al.[55] | 2013 | Meta-analysis –  20 RCTs **(THA)** | - significant reduction of patients needing dressing reinforcement - significantly higher need for blood transfusion - no effect on possibility of infection, loss of blood, drop of Hemoglobin and Hematokrit, functional assessment - no routine use in elective THA |  |
| Tian et al.[56] | 2013 | Meta-analysis –  17 RCTs **(THA)** | - significantly higher percentage of patients requiring blood transfusion - significant increase in blood loss and LOS - no significant effect on complications - no support of routine use in THA |  |
| Nanni et al.[57] | 2013 | Systematic review –  9 studies **(THA)** | - no advantages of drainages after THA found, possibility of complications due to greater need for blood transfusion in reviewed studies |  |
| Zhang et al.[58] | 2012 | Meta-analysis –  21 RCTs **(TKA)** | - significant decrease concerning soft tissue ecchymosis - significant increase in loss of blood followed by higher need for homologous blood transfusion - no effect on ROM, infection rate or DVT - no obvious advantages of drains in TKA |  |

Fig. E. Variation in drainage use at surgeon level (TKA)

Surgeons

Percentage of patients who received drains (TKA)

Percentage and 95% CI of patients who received intra-articular drainage per surgeon. Each filled square represents a single surgeon. The size of the filled square represents the relative number of patients operated on by each surgeon. Surgeons who operated on less than two patients were excluded.

Fig. F. Variation in drainage use at surgeon level (THA)

Surgeons

Percentage of patients who received drains (THA)

Percentage and 95% CI of patients who received intra-articular drainage per surgeon. Each filled square represents a single surgeon. The size of the filled square represents the relative number of patients operated on by each surgeon

# Antibiotic loaded bone cement (ALBC) – Rationale

Surgical site infection (SSI) is a major complication following arthroplasty. Although the perioperative use of systemic antibiotics is the standard [59, 60], the risk of infection remains at around 1% after hip arthroplasty [61, 62] and 1-3% after knee arthroplasty [62, 63]. Periprosthetic infection often requires revision surgery and is thereby associated with higher costs as well as a prolonged hospitalization and can possibly result in a worse functional outcome and level of patient satisfaction [61, 64, 65].

Antibiotic loaded bone cement, first described by Buchholz et al.[66], was a concept conceived to reduce mainly deep surgical infections following TKA or THA.

Considering the unclear benefit on prevention of SSI and the possible adverse effects such as impacts on the bone mechanics, potential toxicity, allergic reactions and development of antibiotic resistances [67-69], the routine use of ALBC in primary knee and hip arthroplasty is questioned.

Table E summarises the conclusions from related systematic reviews and meta-analyses

Figure G shows variation in percentage of patients who received ALBC following TKA per surgeon.

Figure H shows variation in percentage of patients who received ALBC following THA per surgeon.

Fig G. Variation in ALBC use at surgeon level (TKA)

Percentage of patients who received antibiotic loaded bone

cement (TKA)

Surgeons

Percentage and 95% CI of patients who received ALBC per surgeon. Each filled square represents a single surgeon. The size of the filled square represents the relative number of patients operated on by each surgeon. Surgeons who operated on less than two patients were excluded.

Fig H. Variation in ALBC use at surgeon level (THA)

Percentage of patients who received antibiotic loaded bone

cement (THA)

Surgeons

Percentage and 95% CI of patients who received ALBC per surgeon. Each filled square represents a single surgeon. The size of the filled square represents the relative number of patients operated on by each surgeon. Surgeons who operated on less than two patients were excluded.

## *Table E - Antibiotic cement in TKA and THA (compared to non-antibiotic cement if not specified)*

| Study | Year of publication | Study type | Conclusion | |
| --- | --- | --- | --- | --- |
| Zhou et al.[70] | 2015 | Meta-analysis –  three RCTs, one prospective comparative and one retrospective comparative trial **(TKA)** | - no significant reduction of superficial infections - no signification reduction of deep infections - no adverse effects observed after use of ALBC (Antibiotic loaded bone cement) - no efficacy of routine use in primary TKA |  |
| Yi et al.[71] | 2014 | Meta-analysis –  four RCTs and two retrospective trials (four studies **TKA**, one study **THA**, one study TSA) | - no reduction of incidence of infections |  |
| Wang et al.[60] | 2013 | Meta-analysis –  eight RCTSs **(TKA, THA)** | Superficial infections:   - significant reduction when using SA (systematic antibiotics) compared to ALBC - no differences when comparing ALBC to non-antibiotic cement   Deep infections:   - significant reduction when using ALBC compared to SA - significantly lower when using ALBC compared to control in THA - no differences when using ALBC compared to control in TKA - patient safety not compromised |  |

# Patellar resurfacing – Rationale

Anterior knee pain (AKP) is a potential consequence following TKA. Is has been shown that 7 to 47% of patients that did not receive patellar resurfacing during TKA report AKP [72-75]. The risk of postoperative AKP seems to be reduced when the patella is resurfaced [76-78]. However, there are several complications such as patellar fracture, instability, avascular necrosis and patellar tendon injury [79, 80] associated with patella resurfacing. Currently, there are three approaches the surgeon may take:

Routinely non-resurfacing; routinely resurfacing, and; resurfacing based on specific pre- and intraoperatively identified criteria, for example BMI, height, cartilage status or patellar shape.

Table F summarises the conclusions from related systematic reviews and meta-analyses.

Figure I shows variation in percentage of patients who received patellar resurfacing per surgeon.

Fig I. Variation in patellar resurfacing at surgeon level

Percentage of patients who received patellar resurfacing

Surgeons

Percentage and 95% CI of patients with patellar resurfacing per surgeon. Each filled square represents a single surgeon. The size of the filled square represents the relative number of patients operated on by each surgeon. Surgeons who operated on less than two patients were excluded.

## *Table F - Patellar resurfacing in TKA (compared to non-resurfacing if not specified)*

| Study | Year of Publication | Study Type | Conclusion |
| --- | --- | --- | --- |
| Arirachakaran et al.[81] | 2015 | Meta-analysis –  15 RCTs or quasi-experimental studies (concerning patella-resurfacing) | - no significant differences in pain, functional outcome, anterior knee pain or patellofemoral problems - significant decrease of reoperation risk |
| Antholz et al.[82] | 2015 | Systematic review –  41 manuscripts (including SR, meta-analyses, RCTs, non-RCTs and registry data) | - after non-resurfacing higher reoperation risk - routinely resurfacing the patella has an acceptable outcome - individual surgeon’s decision (including pre- and intraoperative aspects) is a reasonable option |
| Nikolaou et al.[12] | 2014 | Systematic review – five meta-analyses and five RCTs | - no support of routine use although reduction of reoperation rate |
| Chen et al.[83] | 2013 | Meta-analysis –  14 RCTs | - significant reduction of reoperation risk - significant increase in Knee Society Score (only in >5y. follow-up) - no significant effect on anterior knee pain or knee pain score and functional scores (overall) |
| Pilling et al.[84] | 2012 | Meta-analysis –  16 RCTs | - no significant effect on functional outcome, anterior knee pain, satisfaction of outcome, infection and operation time - significant higher result in Knee Society Score - significant lower rate of reoperation and patellofemoral complications |
| Bao et al.[85] | 2012 | Meta-analysis –  13 RCTs | - significant decrease of anterior knee pain - no significant effect on knee score and functional scores |
| Pavlou et al.[86] | 2011 | Meta-analysis –  18 RCTs | - no significant effect on postoperative functional outcome and anterior knee pain - significantly lower reoperation rate |
| Li et al.[87] | 2011 | Meta-analysis –  16 RCTs | - no effect on reoperation rate after less than five years - significant lower reoperation rate in one to ten years and five and more years follow-up - no significant effect on functional scores, knee score, patient satisfaction and anterior knee pain (up to five years, up to ten years and five and more years follow-up) - insufficient evidence for routinely resurfacing the patella |
| He et al.[88] | 2011 | Meta-analysis –  17 RCTs | - no significant effect on anterior knee pain, knee pain score, knee society score, knee function score,   or reoperation rate (if five best-quality studies included)   - significantly lower reoperation rate (if 13 studies included) |
| Fu et al.[89] | 2011 | Meta-analysis –  10 RCTs | - no significant effect on anterior knee pain, functional scores or knee score - significantly lower risk of reoperation (due to patellofemoral complications) - non-resurfacing reasonable option (with risk of reoperation) |
| Agrawal et al.[90] | 2011 | Meta-analysis –  16 RCTs | - significant reduction concerning reoperation rate and occurrence of anterior knee pain (including all studies) - no significant effect on anterior knee pain (only high-quality studies) or functional scores |

**Indwelling Urinary Catheters – Rationale**

Urinary catheters are inserted prior to surgery with the aim of preventing post-operative urinary retention (POUR), urinary infection and discomfort. Different factors increasing the risk of POUR have been identified, including increasing age[91], male gender[91], type of surgery[91], past history of urologic problems[92], operation time [93] and the use of certain medications like opioids[93], especially via epidural or spinal anaesthetic (21). The reported incidence of urinary retention following TKA or THA varies widely from 0 to 70%.[94]. Urinary tract infections are also commonly observed after these surgeries (23) and are reported to occur in 32%[94].

Table G summarises the conclusions from related systematic reviews and meta-analyses.

Figure J shows variation in percentage of patients who received an IDC following TKA and THA per surgeon.

Fig J. Variation in IDC use at surgeon level

Surgeons

Percentage of patients who received IDC

Percentage and 95% CI of patients who received IDC per surgeon. Each filled square represents a single surgeon. The size of the filled square represents the relative number of patients operated on by each surgeon. Surgeons who operated on less than two patients were excluded.

*Table G - Indwelling Catheter in TKA and THA (compared to intermittent catheterization)*

| Study | Year of publication | Study Type | Conclusion |
| --- | --- | --- | --- |
| Zhang et al.[93] | 2015 | Meta-analysis –  nine RCTs | - no significant effect on occurrence of urinary tract infection - significant lower rate of postoperative urinary retention (POUR) - in high-risk of POUR patients routine use of IDC recommended |

# *Figure K – Literature Search Example*

**Cryotherapy**

Database: Ovid MEDLINE(R) 1946 to Present with Daily Update

Search Strategy:

--------------------------------------------------------------------------------

1 exp Cryotherapy/ (22364)

2 exp "Review"/ (2040819)

3 exp Meta-Analysis/ (64461)

4 exp Guideline/ (27470)

5 exp Arthroplasty, Replacement, Knee/ or exp Arthroplasty, Replacement, Hip/ (31639)

6 1 and 2 and 5 (7)

7 1 and 4 and 5 (0)

8 1 and 3 and 5 (2)

Database: Embase Classic+Embase <1947 to 2016 April 15>

Search Strategy:

--------------------------------------------------------------------------------

1 exp cryotherapy/ (28494)

2 exp hip arthroplasty/ or exp knee arthroplasty/ (43207)

3 meta-analysis/ (107659)

4 guideline .mp. (302775)

5 "review"/ (2120250)

6 1 and 2 and 5 (28)

7 1 and 2 and 4 (0)

8 1 and 2 and 3 (2)

PubMed: “cryotherapy arthroplasty knee hip review”

PubMed: “cryotherapy arthroplasty knee hip guideline”

PubMed: “cryotherapy arthroplasty knee hip meta-analysis”

**References**

1. Brosseau L, Milne S, Wells G, Tugwell P, Robinson V, Casimiro L, et al. Efficacy of continuous passive motion following total knee arthroplasty: a metaanalysis. J Rheumatol. 2004;31(11):2251-64. PubMed PMID: 15517640.

2. Milne S, Brosseau L, Robinson V, Noel MJ, Davis J, Drouin H, et al. Continuous passive motion following total knee arthroplasty. The Cochrane database of systematic reviews. 2003;(2):CD004260. doi: 10.1002/14651858.CD004260. PubMed PMID: 12804511.

3. Rowe PJ, Myles CM, Walker C, Nutton R. Knee joint kinematics in gait and other functional activities measured using flexible electrogoniometry: how much knee motion is sufficient for normal daily life? Gait Posture. 2000;12(2):143-55. PubMed PMID: 10998612.

4. Kim J, Nelson CL, Lotke PA. Stiffness after total knee arthroplasty. Prevalence of the complication and outcomes of revision. The Journal of bone and joint surgery American volume. 2004;86-A(7):1479-84. PubMed PMID: 15252096.

5. Naylor JM, Ko V, Rougellis S, Green N, Hackett D, Magrath A, et al. Is discharge knee range of motion a useful and relevant clinical indicator after total knee replacement? Part 1. J Eval Clin Pract. 2012;18(3):644-51. doi: 10.1111/j.1365-2753.2011.01655.x. PubMed PMID: 21414107.

6. McCarthy MR, O'Donoghue PC, Yates CK, Yates-McCarthy JL. The clinical use of continuous passive motion in physical therapy. J Orthop Sports Phys Ther. 1992;15(3):132-40. doi: 10.2519/jospt.1992.15.3.132. PubMed PMID: 18796785.

7. Sheppard M, Westlake SM, McQuarrie A. Continuous passive motion - where are we now? Physiotherapy Canada. 1995;47:36-9.

8. Salter RB, Simmonds DF, Malcolm BW, Rumble EJ, MacMichael D, Clements ND. The biological effect of continuous passive motion on the healing of full-thickness defects in articular cartilage. An experimental investigation in the rabbit. The Journal of bone and joint surgery American volume. 1980;62(8):1232-51. PubMed PMID: 7440603.

9. Naylor J, Harmer A, Fransen M, Crosbie J, Innes L. Status of physiotherapy rehabilitation after total knee replacement in Australia. Physiother Res Int. 2006;11(1):35-47. PubMed PMID: 16594314.

10. Association APT. Five things physical therapists and patients should question. 2015. Available from:

http://www.choosingwisely.org/societies/american-physical-therapy-association/

11. Sanchez Mayo B, Rodriguez-Mansilla J, Gonzalez Sanchez B. [Recovery from total knee arthroplasty through continuous passive motion]. An Sist Sanit Navar. 2015;38(2):297-310. PubMed PMID: 26486536.

12. Nikolaou VS, Chytas D, Babis GC. Common controversies in total knee replacement surgery: Current evidence. World J Orthop. 2014;5(4):460-8. doi: 10.5312/wjo.v5.i4.460. PubMed PMID: 25232522; PubMed Central PMCID: PMCPMC4133452.

13. Harvey LA, Brosseau L, Herbert RD. Continuous passive motion following total knee arthroplasty in people with arthritis. The Cochrane database of systematic reviews. 2010;(3):CD004260. doi: 10.1002/14651858.CD004260.pub2. PubMed PMID: 20238330.

14. Harvey LA, Brosseau L, Herbert RD. Continuous passive motion following total knee arthroplasty in people with arthritis. The Cochrane database of systematic reviews. 2014;2:CD004260. doi: 10.1002/14651858.CD004260.pub3. PubMed PMID: 24500904.

15. Yip S.M. TY, Toh J.Y., Phua J.Y., Goh S.P., Ismail M.H. . The effect of continuous passive motion on range of motion after total knee arthroplasty-a systematic review. SingHealth DukeNUS Scientific Congress 2010 Singapore Singapore Conference Start: 20101015 Conference End: 20101015. 2010.

16. Hoylaerts M, Lijnen HR, Collen D. Studies on the mechanism of the antifibrinolytic action of tranexamic acid. Biochim Biophys Acta. 1981;673(1):75-85. PubMed PMID: 7193484.

17. Dietrich W, Spannagl M, Boehm J, Hauner K, Braun S, Schuster T, et al. Tranexamic acid and aprotinin in primary cardiac operations: an analysis of 220 cardiac surgical patients treated with tranexamic acid or aprotinin. Anesth Analg. 2008;107(5):1469-78. doi: 10.1213/ane.0b013e318182252b. PubMed PMID: 18931201.

18. Coetzee MJ. The use of topical crushed tranexamic acid tablets to control bleeding after dental surgery and from skin ulcers in haemophilia. Haemophilia. 2007;13(4):443-4. doi: 10.1111/j.1365-2516.2007.01479.x. PubMed PMID: 17610565.

19. Rannikko A, Petas A, Taari K. Tranexamic acid in control of primary hemorrhage during transurethral prostatectomy. Urology. 2004;64(5):955-8. doi: 10.1016/j.urology.2004.07.008. PubMed PMID: 15533485.

20. Ralley FE, Berta D, Binns V, Howard J, Naudie DD. One intraoperative dose of tranexamic Acid for patients having primary hip or knee arthroplasty. Clin Orthop Relat Res. 2010;468(7):1905-11. doi: 10.1007/s11999-009-1217-8. PubMed PMID: 20063079; PubMed Central PMCID: PMCPMC2882010.

21. Benoni G, Fredin H. Fibrinolytic inhibition with tranexamic acid reduces blood loss and blood transfusion after knee arthroplasty: a prospective, randomised, double-blind study of 86 patients. J Bone Joint Surg Br. 1996;78(3):434-40. PubMed PMID: 8636182.

22. Good L, Peterson E, Lisander B. Tranexamic acid decreases external blood loss but not hidden blood loss in total knee replacement. Br J Anaesth. 2003;90(5):596-9. PubMed PMID: 12697586.

23. Chen S, Wu K, Kong G, Feng W, Deng Z, Wang H. The efficacy of topical tranexamic acid in total hip arthroplasty: a meta-analysis. BMC Musculoskelet Disord. 2016;17(1):81. doi: 10.1186/s12891-016-0923-0. PubMed PMID: 26878845; PubMed Central PMCID: PMCPMC4754977.

24. Wei Z, Liu M. The effectiveness and safety of tranexamic acid in total hip or knee arthroplasty: a meta-analysis of 2720 cases. Transfus Med. 2015;25(3):151-62. doi: 10.1111/tme.12212. PubMed PMID: 26033447.

25. Xu X, Xiong S, Wang Z, Li X, Liu W. Topical administration of tranexamic acid in total hip arthroplasty: A meta-analysis of Randomized Controlled Trials. Drug Discov Ther. 2015;9(3):173-7. doi: 10.5582/ddt.2015.01018. PubMed PMID: 26193938.

26. Yue C, Pei F, Yang P, Xie J, Kang P. Effect of Topical Tranexamic Acid in Reducing Bleeding and Transfusions in TKA. Orthopedics. 2015;38(5):315-24. doi: 10.3928/01477447-20150504-06. PubMed PMID: 25970359.

27. Wu Q; Zhang HA; Liu SL; Meng T; Zhou X; Wang P. Is tranexamic acid clinically effective and safe to prevent blood loss in total knee arthroplasty? A meta-analysis of 34 randomized controlled trials. [Review]. European journal of orthopaedic surgery & traumatologie. 2015;25(3):525-41.

28. Shemshaki H, Nourian SM, Nourian N, Dehghani M, Mokhtari M, Mazoochian F. One step closer to sparing total blood loss and transfusion rate in total knee arthroplasty: a meta-analysis of different methods of tranexamic acid administration. Arch Orthop Trauma Surg. 2015;135(4):573-88. doi: 10.1007/s00402-015-2189-7. PubMed PMID: 25739992.

29. Wang C, Xu GJ, Han Z, Ma JX, Ma XL, Jiang X, et al. Topical application of tranexamic acid in primary total hip arthroplasty: a systemic review and meta-analysis. Int J Surg. 2015;15:134-9. doi: 10.1016/j.ijsu.2014.12.023. PubMed PMID: 25576011.

30. Zhang Y, Fu X, Liu WX, Li YM, Ma XL, Li ZJ. Safety and efficacy of intra-articular injection of tranexamic acid in total knee arthroplasty. Orthopedics. 2014;37(9):e775-82. doi: 10.3928/01477447-20140825-53. PubMed PMID: 25350619.

31. Zhao-Yu C, Yan G, Wei C, Yuejv L, Ying-Ze Z. Reduced blood loss after intra-articular tranexamic acid injection during total knee arthroplasty: a meta-analysis of the literature. Knee Surg Sports Traumatol Arthrosc. 2014;22(12):3181-90. doi: 10.1007/s00167-013-2814-3. PubMed PMID: 24352523.

32. Wang H, Shen B, Zeng Y. Comparison of topical versus intravenous tranexamic acid in primary total knee arthroplasty: a meta-analysis of randomized controlled and prospective cohort trials. Knee. 2014;21(6):987-93. doi: 10.1016/j.knee.2014.09.010. PubMed PMID: 25450009.

33. Alshryda S, Sukeik M, Sarda P, Blenkinsopp J, Haddad FS, Mason JM. A systematic review and meta-analysis of the topical administration of tranexamic acid in total hip and knee replacement. Bone Joint J. 2014;96-B(8):1005-15. doi: 10.1302/0301-620X.96B8.33745. PubMed PMID: 25086114.

34. Panteli M, Papakostidis C, Dahabreh Z, Giannoudis PV. Topical tranexamic acid in total knee replacement: a systematic review and meta-analysis. Knee. 2013;20(5):300-9. doi: 10.1016/j.knee.2013.05.014. PubMed PMID: 23815893.

35. Gandhi R, Evans HM, Mahomed SR, Mahomed NN. Tranexamic acid and the reduction of blood loss in total knee and hip arthroplasty: a meta-analysis. BMC Res Notes. 2013;6:184. doi: 10.1186/1756-0500-6-184. PubMed PMID: 23651507; PubMed Central PMCID: PMCPMC3655041.

36. Fu DJC, C. Guo, L. Yang, L. Use of intravenous tranexamic acid in total knee arthroplasty: a meta-analysis of randomized controlled trials. Chin J Traumatol. 2013;16(2):67-76. PubMed PMID: 23540893.

37. Zhang H, Chen J, Chen F, Que W. The effect of tranexamic acid on blood loss and use of blood products in total knee arthroplasty: a meta-analysis. Knee Surg Sports Traumatol Arthrosc. 2012;20(9):1742-52. doi: 10.1007/s00167-011-1754-z. PubMed PMID: 22065294.

38. Sukeik M, Alshryda S, Haddad FS, Mason JM. Systematic review and meta-analysis of the use of tranexamic acid in total hip replacement. J Bone Joint Surg Br. 2011;93(1):39-46. doi: 10.1302/0301-620X.93B1.24984. PubMed PMID: 21196541.

39. Adie S, Naylor JM, Harris IA. Cryotherapy after total knee arthroplasty a systematic review and meta-analysis of randomized controlled trials. J Arthroplasty. 2010;25(5):709-15. PubMed PMID: 19729279.

40. Dundon JM, Rymer MC, Johnson RM. Total patellar skin loss from cryotherapy after total knee arthroplasty. J Arthroplasty. 2013;28(2):376 e5-7. doi: 10.1016/j.arth.2012.05.024. PubMed PMID: 22749659.

41. Gibbons CE, Solan MC, Ricketts DM, Patterson M. Cryotherapy compared with Robert Jones bandage after total knee replacement: a prospective randomized trial. Int Orthop. 2001;25(4):250-2. PubMed PMID: 11561502; PubMed Central PMCID: PMCPMC3620829.

42. Ni SH, Jiang WT, Guo L, Jin YH, Jiang TL, Zhao Y, et al. Cryotherapy on postoperative rehabilitation of joint arthroplasty. Knee Surg Sports Traumatol Arthrosc. 2015;23(11):3354-61. doi: 10.1007/s00167-014-3135-x. PubMed PMID: 24928371.

43. Adie S, Kwan A, Naylor JM, Harris IA, Mittal R. Cryotherapy following total knee replacement. Cochrane Database of Systematic Reviews. 2012;9:CD007911. PubMed PMID: 22972114.

44. Markert SE. The use of cryotherapy after a total knee replacement: a literature review. Orthop Nurs. 2011;30(1):29-36. PubMed PMID: 21278552.

45. Alexander JW, Korelitz J, Alexander NS. Prevention of wound infections. A case for closed suction drainage to remove wound fluids deficient in opsonic proteins. Am J Surg. 1976;132(1):59-63. PubMed PMID: 782267.

46. Waugh TR, Stinchfield FE. Suction drainage of orthopaedic wounds. The Journal of bone and joint surgery American volume. 1961;43-A:939-46. PubMed PMID: 14040185.

47. Quinn M, Bowe A, Galvin R, Dawson P, O'Byrne J. The use of postoperative suction drainage in total knee arthroplasty: a systematic review. Int Orthop. 2015;39(4):653-8. doi: 10.1007/s00264-014-2455-2. PubMed PMID: 25027980.

48. Chen ZY, Gao Y, Chen W, Li X, Zhang YZ. Is wound drainage necessary in hip arthroplasty? A meta-analysis of randomized controlled trials. Eur J Orthop Surg Traumatol. 2014;24(6):939-46. doi: 10.1007/s00590-013-1284-0. PubMed PMID: 23917702.

49. Parker MJ, Livingstone V, Clifton R, McKee A. Closed suction surgical wound drainage after orthopaedic surgery. The Cochrane database of systematic reviews. 2007;(3):CD001825. doi: 10.1002/14651858.CD001825.pub2. PubMed PMID: 17636687.

50. Cheung G, Carmont MR, Bing AJ, Kuiper JH, Alcock RJ, Graham NM. No drain, autologous transfusion drain or suction drain? A randomised prospective study in total hip replacement surgery of 168 patients. Acta Orthop Belg. 2010;76(5):619-27. PubMed PMID: 21138217.

51. Dora C, von Campe A, Mengiardi B, Koch P, Vienne P. Simplified wound care and earlier wound recovery without closed suction drainage in elective total hip arthroplasty. A prospective randomized trial in 100 operations. Arch Orthop Trauma Surg. 2007;127(10):919-23. doi: 10.1007/s00402-006-0260-0. PubMed PMID: 17165035.

52. Gonzalez Della Valle A, Slullitel G, Vestri R, Comba F, Buttaro M, Piccaluga F. No need for routine closed suction drainage in elective arthroplasty of the hip: a prospective randomized trial in 104 operations. Acta Orthop Scand. 2004;75(1):30-3. doi: 10.1080/00016470410001708050. PubMed PMID: 15022802.

53. Zan P. WW, Fan L., Wu Z., Yu X., Xu T., Li G. . Closed-suction drainage versus no drainage in total hip arthroplasty, a meta-analysis of randomized controlled trials. International Journal of Clinical and Experimental Medicine. 2016;9((2)):725-35.

54. Kelly EG, Cashman JP, Imran FH, Conroy R, O'Byrne J. Systematic review and meta-analysis of closed suction drainage versus non-drainage in primary hip arthroplasty. Surg Technol Int. 2014;24:295-301. PubMed PMID: 24574017.

55. Zhou XD, Li J, Xiong Y, Jiang LF, Li WJ, Wu LD. Do we really need closed-suction drainage in total hip arthroplasty? A meta-analysis. Int Orthop. 2013;37(11):2109-18. doi: 10.1007/s00264-013-2053-8. PubMed PMID: 23982636; PubMed Central PMCID: PMCPMC3824906.

56. Tian R.-Y. YP, Deng J., Huang W.-L., Ma L.-K., Lu X.-F. Systematic review on the effect of drainage on the rehabilitation of the patients after total hip arthroplasty. Chinese Journal of Tissue Engineering Research. 2013;17((35)):6300-5.

57. Nanni M, Perna F, Calamelli C, Donati D, Ferrara O, Parlato A, et al. Wound drainages in total hip arthroplasty: to use or not to use? Review of the literature on current practice. Musculoskelet Surg. 2013;97(2):101-7. doi: 10.1007/s12306-013-0270-3. PubMed PMID: 23709186.

58. Zhang XN, Wu G, Xu RZ, Bai XZ. [Closed suction drainage or non-drainage for total knee arthroplasty: a meta-analysis]. Zhonghua Wai Ke Za Zhi. 2012;50(12):1119-25. PubMed PMID: 23336492.

59. Hinarejos P, Guirro P, Puig-Verdie L, Torres-Claramunt R, Leal-Blanquet J, Sanchez-Soler J, et al. Use of antibiotic-loaded cement in total knee arthroplasty. World J Orthop. 2015;6(11):877-85. doi: 10.5312/wjo.v6.i11.877. PubMed PMID: 26716084; PubMed Central PMCID: PMCPMC4686435.

60. Wang J, Zhu C, Cheng T, Peng X, Zhang W, Qin H, et al. A systematic review and meta-analysis of antibiotic-impregnated bone cement use in primary total hip or knee arthroplasty. PLoS One. 2013;8(12):e82745. doi: 10.1371/journal.pone.0082745. PubMed PMID: 24349353; PubMed Central PMCID: PMCPMC3861452.

61. Zimmerli W, Trampuz A, Ochsner PE. Prosthetic-joint infections. N Engl J Med. 2004;351(16):1645-54. doi: 10.1056/NEJMra040181. PubMed PMID: 15483283.

62. Kurtz SM, Lau E, Schmier J, Ong KL, Zhao K, Parvizi J. Infection burden for hip and knee arthroplasty in the United States. J Arthroplasty. 2008;23(7):984-91. doi: 10.1016/j.arth.2007.10.017. PubMed PMID: 18534466.

63. Cram P, Lu X, Kates SL, Singh JA, Li Y, Wolf BR. Total knee arthroplasty volume, utilization, and outcomes among Medicare beneficiaries, 1991-2010. JAMA. 2012;308(12):1227-36. doi: 10.1001/2012.jama.11153. PubMed PMID: 23011713; PubMed Central PMCID: PMCPMC4169369.

64. Dunbar MJ. Antibiotic bone cements: their use in routine primary total joint arthroplasty is justified. Orthopedics. 2009;32(9). doi: 10.3928/01477447-20090728-20. PubMed PMID: 19751021.

65. Department of Orthopedics LUH. The Swedish Knee Arthroplasty Annual Report 2008. 2008.

66. Buchholz HW, Engelbrecht H. [Depot effects of various antibiotics mixed with Palacos resins]. Chirurg. 1970;41(11):511-5. PubMed PMID: 5487941.

67. Hanssen AD. Prophylactic use of antibiotic bone cement: an emerging standard--in opposition. J Arthroplasty. 2004;19(4 Suppl 1):73-7. PubMed PMID: 15190554.

68. Joseph TN, Chen AL, Di Cesare PE. Use of antibiotic-impregnated cement in total joint arthroplasty. J Am Acad Orthop Surg. 2003;11(1):38-47. PubMed PMID: 12699370.

69. Arciola CR, Campoccia D, Montanaro L. Effects on antibiotic resistance of Staphylococcus epidermidis following adhesion to polymethylmethacrylate and to silicone surfaces. Biomaterials. 2002;23(6):1495-502. PubMed PMID: 11829446.

70. Zhou Y, Li L, Zhou Q, Yuan S, Wu Y, Zhao H, et al. Lack of efficacy of prophylactic application of antibiotic-loaded bone cement for prevention of infection in primary total knee arthroplasty: results of a meta-analysis. Surg Infect (Larchmt). 2015;16(2):183-7. doi: 10.1089/sur.2014.044. PubMed PMID: 25826289.

71. Yi Z, Bin S, Jing Y, Zongke Z, Pengde K, Fuxing P. No decreased infection rate when using antibiotic-impregnated cement in primary total joint arthroplasty. Orthopedics. 2014;37(12):839-45. doi: 10.3928/01477447-20141124-07. PubMed PMID: 25437076.

72. Levitsky KA, Harris WJ, McManus J, Scott RD. Total knee arthroplasty without patellar resurfacing. Clinical outcomes and long-term follow-up evaluation. Clin Orthop Relat Res. 1993;(286):116-21. PubMed PMID: 8425332.

73. Fern ED, Winson IG, Getty CJ. Anterior knee pain in rheumatoid patients after total knee replacement. Possible selection criteria for patellar resurfacing. J Bone Joint Surg Br. 1992;74(5):745-8. PubMed PMID: 1527126.

74. Lyback CO, Lehto MU, Hamalainen MM, Belt EA. Patellar resurfacing reduces pain after TKA for juvenile rheumatoid arthritis. Clin Orthop Relat Res. 2004;(423):152-6. PubMed PMID: 15232441.

75. Boyd AD, Jr., Ewald FC, Thomas WH, Poss R, Sledge CB. Long-term complications after total knee arthroplasty with or without resurfacing of the patella. The Journal of bone and joint surgery American volume. 1993;75(5):674-81. PubMed PMID: 8501082.

76. Patel K, Raut V. Patella in total knee arthroplasty: to resurface or not to--a cohort study of staged bilateral total knee arthroplasty. Int Orthop. 2011;35(3):349-53. doi: 10.1007/s00264-010-1063-z. PubMed PMID: 20559832; PubMed Central PMCID: PMCPMC3047646.

77. Clements WJ, Miller L, Whitehouse SL, Graves SE, Ryan P, Crawford RW. Early outcomes of patella resurfacing in total knee arthroplasty. Acta Orthop. 2010;81(1):108-13. doi: 10.3109/17453670903413145. PubMed PMID: 19968604; PubMed Central PMCID: PMCPMC2856213.

78. Badhe N, Dewnany G, Livesley PJ. Should the patella be replaced in total knee replacement? Int Orthop. 2001;25(2):97-9. PubMed PMID: 11409461; PubMed Central PMCID: PMCPMC3620637.

79. Ortiguera CJ, Berry DJ. Patellar fracture after total knee arthroplasty. The Journal of bone and joint surgery American volume. 2002;84-A(4):532-40. PubMed PMID: 11940611.

80. Keblish PA, Varma AK, Greenwald AS. Patellar resurfacing or retention in total knee arthroplasty. A prospective study of patients with bilateral replacements. J Bone Joint Surg Br. 1994;76(6):930-7. PubMed PMID: 7983122.

81. Arirachakaran A, Sangkaew C, Kongtharvonskul J. Patellofemoral resurfacing and patellar denervation in primary total knee arthroplasty. Knee Surg Sports Traumatol Arthrosc. 2015;23(6):1770-81. doi: 10.1007/s00167-014-3311-z. PubMed PMID: 25218579.

82. Antholz CR, Cherian JJ, Elmallah RK, Jauregui JJ, Pierce TP, Mont MA. Selective Patellar Resurfacing: A Literature Review. Surg Technol Int. 2015;26:355-60. PubMed PMID: 26055032.

83. Chen K, Li G, Fu D, Yuan C, Zhang Q, Cai Z. Patellar resurfacing versus nonresurfacing in total knee arthroplasty: a meta-analysis of randomised controlled trials. Int Orthop. 2013;37(6):1075-83. doi: 10.1007/s00264-013-1866-9. PubMed PMID: 23529719; PubMed Central PMCID: PMCPMC3664152.

84. Pilling RW, Moulder E, Allgar V, Messner J, Sun Z, Mohsen A. Patellar resurfacing in primary total knee replacement: a meta-analysis. The Journal of bone and joint surgery American volume. 2012;94(24):2270-8. doi: 10.2106/JBJS.K.01257. PubMed PMID: 23318618.

85. Bao L, Hu JH, Jin QH. [A analysis on the effect of with or without patellar replacement in total knee arthroplasty]. Zhonghua Wai Ke Za Zhi. 2012;50(2):171-5. PubMed PMID: 22490360.

86. Pavlou G, Meyer C, Leonidou A, As-Sultany M, West R, Tsiridis E. Patellar resurfacing in total knee arthroplasty: does design matter? A meta-analysis of 7075 cases. The Journal of bone and joint surgery American volume. 2011;93(14):1301-9. doi: 10.2106/JBJS.J.00594. PubMed PMID: 21792496.

87. Li S, Chen Y, Su W, Zhao J, He S, Luo X. Systematic review of patellar resurfacing in total knee arthroplasty. Int Orthop. 2011;35(3):305-16. doi: 10.1007/s00264-010-1109-2. PubMed PMID: 20803354; PubMed Central PMCID: PMCPMC3047648.

88. He JY, Jiang LS, Dai LY. Is patellar resurfacing superior than nonresurfacing in total knee arthroplasty? A meta-analysis of randomized trials. Knee. 2011;18(3):137-44. doi: 10.1016/j.knee.2010.04.004. PubMed PMID: 20493712.

89. Fu Y, Wang G, Fu Q. Patellar resurfacing in total knee arthroplasty for osteoarthritis: a meta-analysis. Knee Surg Sports Traumatol Arthrosc. 2011;19(9):1460-6. doi: 10.1007/s00167-010-1365-0. PubMed PMID: 21234539.

90. Agrawal M. JV, Yadav V.P., Bhardwaj V. Patellar resurfacing in total knee arthroplasty. Journal of Clinical Orthopaedics and Trauma. 2011;2((2)):77-82.

91. Tammela T, Kontturi M, Lukkarinen O. Postoperative urinary retention. I. Incidence and predisposing factors. Scand J Urol Nephrol. 1986;20(3):197-201. PubMed PMID: 3787196.

92. Hrubinova J, Hrubina M, Pangrac J. [Urethral catheter as a risk factor of urologic complications after total knee arthoplasty--the retrospective analysis]. Rozhl Chir. 2012;91(1):12-7. PubMed PMID: 22746073.

93. Zhang W, Liu A, Hu D, Xue D, Li C, Zhang K, et al. Indwelling versus Intermittent Urinary Catheterization following Total Joint Arthroplasty: A Systematic Review and Meta-Analysis. PLoS One. 2015;10(7):e0130636. doi: 10.1371/journal.pone.0130636. PubMed PMID: 26146830; PubMed Central PMCID: PMCPMC4492963.

94. Balderi T, Carli F. Urinary retention after total hip and knee arthroplasty. Minerva Anestesiol. 2010;76(2):120-30. PubMed PMID: 20150853.
